# Functional parameters indicative of mild cognitive impairment: a systematic review using instrumented kinematic assessment

**DOI:** 10.1186/s12877-020-01678-6

**Published:** 2020-08-10

**Authors:** Iván José Fuentes-Abolafio, Brendon Stubbs, Luis Miguel Pérez-Belmonte, María Rosa Bernal-López, Ricardo Gómez-Huelgas, Antonio Cuesta-Vargas

**Affiliations:** 1 Department of Physiotherapy, Faculty of Health Science, University ofMálaga. Biomedical Research Institute of Malaga (IBIMA), Clinimetric Group FE-14, Málaga, Spain. Av/ Arquitecto Peñalosa s/n (Teatinos Campus Expansion), 29071 Malaga, Spain; 2 Physiotherapy Department, South London and Maudsley NHS Foundation Trust, Denmark Hill, London, UK; 3 Department of Psychological Medicine, Institute of Psychiatry, Psychology and Neuroscience, King’s College London, London, UK; 4 Positive Ageing Research Intitute (PARI), Faculty of Health Social Care and Education, Anglia Ruskin University, Chelmsford, UK; 5 Internal Medicine Department, Instituto de Investigación Biomédica de Malaga (IBIMA), Regional University Hospital of Málaga, Málaga, Spain; 6 Unidad de Neurofisiología Cognitiva, Centro de Investigaciones Médico Sanitarias (CIMES), Instituto de Investigación Biomédica de Málaga (IBIMA), Universidad de Málaga (UMA), Campus de Excelencia Internacional (CEI) Andalucía Tech, Málaga, Spain; 7 Centro de Investigación Biomédica en Red Enfermedades Cardiovasculares (CIBERCV), Instituto de Salud Carlos III, Madrid, Spain; 8 CIBER Fisio-patología de la Obesidad y la Nutrición, Instituto de Salud Carlos III, Madrid, Spain; 9 School of Clinical Sciences, Faculty of Health at the Queensland University of Technology, Brisbane, Queensland Australia

**Keywords:** Mild cognitive impairment, Functional objective parameters, Instrumented assessment, Kinematics, Gait, Balance

## Abstract

**Background:**

Patients with mild cognitive impairment (MCI) experience alterations of functional parameters, such as an impaired balance or gait. The current systematic review set out to investigate whether functional objective performance may predict a future risk of MCI; to compare functional objective parameters in patients with MCI and a control group; and to assess changes in these parameters after different physical activity interventions.

**Methods:**

Electronic databases, including PubMed, AMED, CINAHL, EMBASE, PEDro and Web of Science as well as grey literature databases, were searched from inception to February 2020. Cohort studies and Randomized Controlled Trials (RCTs) were included. The risk of bias of the included studies was assessed independently by reviewers using quality assessment checklists. The level of evidence per outcome was assessed using the GRADE criteria.

**Results:**

Seventeen studies met inclusion criteria including patients with MCI. Results from RCTs suggested that gait speed, gait variability and balance may be improved by different physical activity interventions. Cohort studies showed that slower gait speed, above all, under Dual Task (DT) conditions, was the main impaired parameter in patients with MCI in comparison with a Control Gorup. Furthermore, cohort studies suggested that gait variability could predict an incident MCI. Although most of included cohort studies reported low risk of bias, RCTs showed an unclear risk of bias.

**Conclusions:**

Studies suggest that gait variability may predict an incident MCI. Moreover, different gait parameters, above all under DT conditions, could be impaired in patients with MCI. These parameters could be improved by some physical activity interventions. Although cohort studies reported low risk of bias, RCTs showed an unclear risk of bias and GRADE criteria showed a low level of evidence per outcome, so further studies are required to refute our findings.

**Prospero:**

CRD42019119180.

## Background

The global life expectancy is increasing in the last years. Consequently, morbidity, chronic individual diseases and the number of people affected by dementia are also increasing [1–5]. Thus, while dementias affected around 46.8 million people worldwide in 2016 [5], it is expected that in 2050 there will be 115–135 million people suffering from dementia [6, 7]. There is an increase in the interest of mild cognitive impairment (MCI), defined as a clinical stage accounting for cognitive impairment that often precedes dementia [5, 8–17], and whose prevalence in adults of ≥65 years old is 10–20%, increasing this prevalence with age [5, 8].

Accepted diagnosis criteria of MCI reported that patients with MCI were characterized by an objective impairment of cognition that is often not severe enough to interfere with activities of daily living (ADL), instrumental activities of daily living (IADL) or in social or occupational functioning [5, 8, 10–14, 17–21]. In the same way, Petersen [11] determined that patients with MCI presented very mild degrees of functional impairment that is difficult to distinguish from the functional problems of cognitively healthy individuals of the same age. However, patients with MCI may have problems in functional tasks [5] and it has been reported that these patients present the alteration of functional parameters, such as mobility, muscle strength, balance, gait dysfunction, or increased risk of falls [8, 22–26]. Slower gait speed has also been suggested as the mainly altered parameter in older populations [23–25, 27–32] which may be a marker for the preclinical stages of dementia [23, 30–33]. Thus, Doi et al. [34], Eggermont et al. [35] and Deshpande et al. [36] reported that slower gait speed could be indicative of MCI. Veronese et al. [30] showed an association between decreased gait speed and low performance in the Short Physical Performance Battery (SPPB) and cognitive decline. Other functional tests, such as Timed Up Go (TUG), Hand Grip Strength Test (HGST), Sit to Stand Test (STS), or Walking Speed ​​Test (WST), have also been used to demonstrate the association between the performance on functional tests and MCI [22, 30, 37, 38].

However, Mirelman et al. [39] reported that patients with MCI could have functional alterations only identifiable through a kinematic analysis conducted in their case with an inertial sensor. In this way, Bahureksa et al. [40] revealed that kinematic gait parameters such as velocity, stride length, and stride time best discriminated patients with MCI from cognitively healthy individuals under single task (ST) conditions and, above all, under dual task (DT) conditions. Balance parameters such as anterior-posterior and medio-lateral sway position also were identified as significant discriminators [40]. Kinematic measurements are frequently used by physicians and researchers to quantify normal and pathological movements and could allow to identify altered objective functional parameters in patients with MCI [41]. The identification of changes in functional objective parameters could be relevant for targeting specific interventions aiming to prevent further functional decline or to improve the functionality of patients with MCI. Currently, no drug has been shown to be effective for MCI [8, 17, 18, 42]. However, it has been reported that the combination of aerobic exercise, balance training, cognitive training, the Mediterranean diet and social commitment could reduce the risk of further cognitive impaiment and may improve cognition, mobility, balance and quality of life in patients with MCI [8, 17, 18, 43, 44]. Considering this, the main objectives of this systematic review were (1) to examine if functional kinematic parameters may predict a future risk of MCI; (2) to compare these functional objective parameters in patients with MCI and a control group; (3) to assess longitudinal changes in these parameters after different physical activity interventions. The secondary objectives were (1) to assess the risk of bias of the included studies using The Newcastle-Ottawa Quality Assessment Scale (NOS) and The Cochrane Collaboration’s tool; (2) to assess the level of evidence per outcome using the Grading of Recommendations Assessment, Development and Evaluation (GRADE).

## Methods

This systematic review was carried out in accordance with the Preferred Reporting Items for Systematic Reviews and Meta-Analyses (PRISMA) statement [45]. The PRISMA checklist for this trial is available in supplementary appendix A. The systematic review protocol was registered at the International Prospective Register of Systematic Reviews (PROSPERO: CRD42019119180).

### Data sources and search strategy

A systematic search was performed by two independent reviewers (IJ-FA and A-CV) from inception to February 2020 using optimised search strategies in the following electronic databases: PubMed, AMED, CINAHL, EMBASE, PEDro, Web of Science. A sensitive search strategy using relevant search terms that were developed from Medical Subject Headings (MeSH), and keywords from other similar studies were used: ‘mild cognitive impaiment’ (MeSH Terms), ‘kinetics’ (MeSH Terms), ‘acceleromet*’ (MeSH Terms), ‘walking speed’ (MeSH Terms), ‘kinematic’, ‘kinematic analysis’, ‘Timed Up and Go’, ‘TUG’, ‘gait speed’, ‘gait speed test’, ‘walking speed test’, ‘short physical performance battery’, ‘SPPB’, ‘six minute walk test’, ‘6 minute walk test’, ‘sit to stand test’, ‘single leg stance test’, ‘one leg stance test’, ‘functional reach test’, ‘romberg test’ and ‘functional task´. The complete search strategy report with all search terms is shown online in supplementary appendix B. The grey literature databases, such as New York Academy of Medicine Grey Literature Report, Grey Literature in Health Research and Open Grey were explored to detect any relevant unpublished data. References were exported, and duplicates were removed using citation management software (Mendeley desktop V.1.19.2).

### Eligibility criteria

Only studies published in full-text papers were included. Abstracts in conference proceedings, poster presentations, notes or letters to the editor were excluded because they had insufficient detail to be evaluated. Each study had to meet the following inclusion criteria:

1. Cohort studies examining the relationship between functional kinematic parameters obtained by instrumented analysis (e.g., electronic walkways, wearable sensors, camera systems …) and incident MCI or comparing these functional objective parameters between confirmed MCI and a Control Group formed by cognitively healthy individuals or people with Alzheimer Disease.
2. RCTs assessing longitudinal changes in functional objective parameters after different physical activity interventions.
3. Studies that included patients with MCI diagnosed by a specialist or which used validated diagnostic criteria (e.g., Petersen’s et al. [11, 12, 14–16], Winblad et al. [13]), supported by a score of 0.5 on the Clinical Dementia Rating (CDR)) [46], < 26 on the Montreal Cognitive Assessment (MoCa) [47, 48], or > 24 Mini-Mental State Examination (MMSE) [48, 49], that permited to confirm the diagnosis of MCI.
4. Studies recruiting participants from any setting (general population, primary, secondary or tertiary care).
5. Studies written in English or Spanish.

The exclusion criteria were as follows:

1. All studies not including a longitudinal design (e.g cross-sectional studies).
2. Studies that included the relationship between functional parameters and incident MCI but did not include a kinematic instrumented analysis.
3. Studies exploring the relationship between functional kinematic parameters and cognitively healthy individuals or people with other neurologic diseases different from MCI.
4. Studies examining the relationship between MCI and other different kinematic parameters such as graphomotor functions, handwriting process variables, etc.
5. Studies that evaluated the relationship between functional kinematic parameters and brain structures in patients with MCI.
6. Studies that did not include validated diagnostic criteria of MCI, did not specify how those patients with MCI were diagnosed or used a diagnosis based on a MMSE score of less than 24, which could be indicative of a greater dementia than the MCI [48–52].

### Study selection

All studies identified by the search strategy were screened using the eligibility criteria previously specified. Two independent reviewers (IJ-FA and A-CV) carried out the first stage, which involved the screening of titles and abstracts to identify potentially relevant records. If the reviewers were unable to determine a study’s eligibility based on title and abstract, the full text was retrieved. In this first stage, the two reviewers also excluded those documents that were not full-text papers. The same reviewers undertook the second stage, screening those articles that met all inclusion criteria. A short checklist was carried out to the present review in order to guide the selection of relevant studies (supplementary appendix C).

### Data extraction

Two independent reviewers (IJ-FA and A-CV) extracted the following relevant data from each study: study details (first author, year of publication), study design, length of follow up, sample size and characteristics of participants (mean age), functional assessment or test used to assess functional parameters, physical activity intervention, instrument used to kinematic analysis and the methods used to diagnose or assess MCI.

### Quality assessment

Two independent reviewers (IJ-FA and A-CV) assessed the risk of bias of the included cohort studies using the NOS [53]. The NOS is a reliable and valid tool for assessing the quality of non-randomized studies [53] and assigns up to a maximum of nine points for the least risk of bias in three domains: selection of study groups (four points); comparability of groups (two points); and ascertainment of exposure and outcomes (three points). The risk of bias of the included RCTs was assessed using The Cochrane Collaboration’s tool [54]. The Cochrane Collaboration’s tool includes seven domain or sources of risk or bias assessment: random sequence generation, allocation concealment, selective reporting, blinding of participants and personnel, blinding of outcome assessment, incomplete outcome data and other bias. For each domain, the risk is categorized as “low risk”, “high risk” or “unclear risk”. To assess the overall quality and the strength of the evidence per outcome, the GRADE approach was used [55]. In brief, the GRADE classification was carried out according to the presence, or not, of the following identified factors: (i) study design, (ii) risk of bias, (iii) inconsistency of results (iv) indirectness (v) imprecision, and (vi) other considerations (e.g. reporting bias). Two researchers (IJ-FA and A-CV) judged whether these factors were present for each outcome. The GRADE criteria was applied when each outcome was informed at least by two studies with the same design. The level of evidence per outcome based on the GRADE criteria is classified as: (1) high (further research is unlikely to change our confidence in the estimate of effect and there are no known or suspected reporting biases); (2) moderate (further research is likely to have an important effect on our confidence in the estimate of effect and might change the estimate); (3) low (further research is likely to have an important effect on our confidence in the estimate of effect and is likely to change the estimate); or (4) very low (we are uncertain about the estimate) [55].

### Data synthesis and analysis

It was planned to conduct a meta-analysis of functional kinematic parameters such as gait, balance, posture or mobility, that could be indicative of MCI. However, due to an observed heterogeneity across studies in the type of design, methods of functional assessment, instruments used to conduct a kinematic analysis, duration of follow-up, statistical analysis, interventions and data presentation, the statistical pooling of results was deemed not appropriate. Therefore, a meta-analysis of results was not conducted, and a descriptive quantitative analysis was carried out. For this reason, a narrative synthesis of the most relevant summary measure and the main change from baseline was reported.

## Results

### Study characteristics

**Figure 1:**
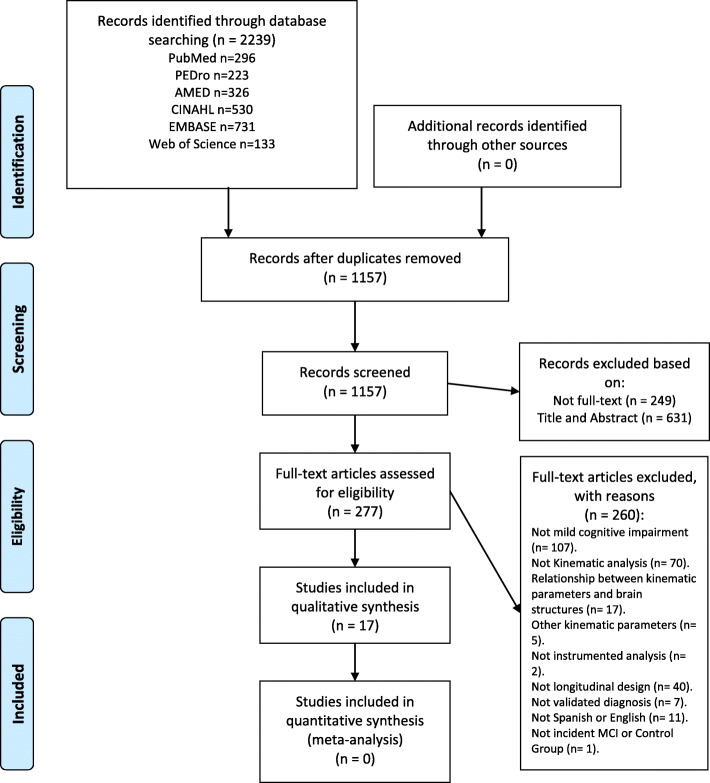
Fig. 1

A total of 2239 citations were identified through electronic databases, with 0 additional studies identified through Grey Literature sources. One thousand one hundred fifty-seven titles and abstracts were screened, and 277 full-text papers were assessed. The number of studies retrieved from each database and the number of studies excluded in each screening phase are shown in Fig. 1. The full reference of excluded studies in the second stage (*n* = 260) is reported online in supplementary appendix D**.** The conflict of interests of included studies are shown online in supplementary appendix E. Of these, 17 studies (six RCTs, one pilot RCT study, one pilot cohort study, eight cohort studies and a reliability study) with a total of 478 participants with MCI and 1540 cognitively healthy individuals at baseline, were included in this review. The characteristics of the included RCTs and the main results are reported in Table 1. The results of cohort studies which compared functional objective parameters between confirmed MCI and a Control Group are reported in Table 2. The characteristics of cohort studies examining the relationship between functional kinematic parameters and incident MCI are showed in Table 3. Functional kinematic parameters were obtained by wearable sensors, tri-axial accelerometers, digital balance platform, motion and contact sensors, cameras and electronic walkways such as the GAITRite (see Table 4). The most frequently used diagnosis criteria of MCI were Petersen criteria (*n* = 7, 41%) and the combination of the CDR (*n* = 9, 53%) and MMSE (*n* = 11, 65%) (see Table 5).

**Table 1:** Summary of included RCT studies involved an Instrumented Functional Assessment as Outcome

Study (first author and year)	Study Design	Study Characteristics (groups, number of participants, mean age)	MCI Diagnostic Criteria	Instrumented Functional Assessment	Instrument	Intervention	Data Collection (follow-up)	Main results in MCI
Doi et al. [56], 2013.	RCT.	Intervention Group: *n* = 25. 75.3 years old. Control Group: n = 25. 76.8 years old.	Petersen Criteria [11]. MMSE > 24 [49].	Walking at preferred speed (11 m walkway).	Tri-axial accelerometer attached to the L3 spinous.	**Intervention Group:** Aerobic exercise (60% of aged predicted maximal heart rate), endurance walking, muscle strength training, postural balance retraining, and gait training (90 min, 2/ week, 6 months). **Control Group**: 2 Education classes about health promotion.	(T1) at baseline; (T2) six months.	↑Gait speed ↓Stride time and ↑Stride length in both groups***. ↑HR in VT in the Intervention group***. ↑Gait speed, stride length and HR in VT in the Intervention group vs control group**.
Donnezan et al. [57], 2018.	RCT.	PCT: *n* = 21. 75.2 years old. PT: *n* = 18. 77.1 years old. CT: *n* = 16. 76.3 years old. Control Group: *n* = 14. 79.2 years old.	Petersen Criteria [11].	Walking speed at usual pace (6 m) in ST and DT conditions. WSC. TUG.	Electronic walkway GAITRite® (length: 4.3 m).	**PT:** Aerobic training on bikes (60% of aged predicted maximal heart rate). **CT:** Cognitive exercises (commercialized gaming software). **PCT:** Aerobic training on bikes (60% of aged predicted maximal heart rate) + cognitive exercises. **Control Group:** Maintaining their usual lifestyle. **All groups:** Two one-hour sessions/week,12 weeks.	(T1) at baseline; (T2) twelve weeks; (T3) six months.	↓Time to perform the TUG***. ↑ Gait speed**. TUG improved after PT and PCT intervention***. Gait speed in ST and DT conditions improved after PCT training***.
Schwenk et al. [58], 2016.	Pilot RCT.	Intervention: *n* = 12. 77.8 years old. Control: *n* = 10. 79.00 years old.	Petersen Criteria [11].	Balance (to stand for 30 s with feet close together with EO and EC. Walking at usual pace and a fast pace (10 m).	Wearable sensors.	**Intervention:** Balance training (weight shifting and virtual obstacle crossing). Real-time visual/ audio lower-limb motion feedback provided from wearable sensors 2/week, 4 weeks). **Control:** No training.	(T1) at baseline; (T2) four weeks.	↓CoM sway in both directions (AP, ML) in the intervention with EO**.
Fogarty et al. [59], 2016.	RCT.	MIP + TTC: *n* = 22. 71.55 years old. MIP: n = 18. 72.61 years old.	Petersen Criteria [11]. MMSE > 24 [49]. MoCA < 26 [47].	Walking at usual pace in ST and DT conditions. CTSIB with EO and EC.	GAITRite® Portable Walkway System. Digital Balance Platform.	**TTC:** Taoist Tai Chi (2/week, 90 min/session, 10 weeks). **MIP:** Education about lifestyle factors that impact memory and teaching of memory strategies (8 sessions).	(T1) at baseline; (T2) ten weeks; (T3) twenty-two weeks.	No significant change between groups in gait variables, the DT cost variables, or in the amount of sway on the balance measures.
Bae et Al. [60], 2018.	RCT.	Intervention: *n* = 41. 75.5 years old. Control: *n* = 42. 76.4 years old.	Winblad Criteria [13]. MMSE > 24 [49].	Maximum hand grip strength. Walking speed and physical activity (time spent in MVPA and step count).	Handheld dynamometer. Tri-axial accelerometer.	**Intervention:** Physical activities (walking, muscle strength training, stretching etc) + cognitive exercises (singing, playing a game, etc) + social activities (2/week, 90 min, 24 weeks). **Control:** 2 Health education classes (90 mins each, during the 24-week).	(T1) at baseline; (T2) six months.	↓Time spent in MVPA after intervention in the control group**. ↓Step count after intervention in the control group**. Intervention Group kept baseline parameters.
Delbroek et al. [61], 2017.	RCT.	Intervention: n = 10. 86.9 years old. Control: n = 10. 87.5 years old.	MoCa < 26 [47].	TUG in ST and DT conditions.	Inertial measurement units on the ankles, wrists and sternum.	**Intervention:** Virtual reality dual-task training using the BioRescue (2/week, 18–30 min, 6 weeks). **Control:** No training.	(T1) at baseline; (T2) six weeks.	↓Total time to perform the TUG in the intervention group during ST condition**.
Liao et al. [62], 2019.	RCT.	Intervention: n = 18. 75.5 years old. Control: n = 16. 73.1 years old.	MoCa < 26 [47].	Walking at preferred Speed in ST and DT conditions.	GAIT Up System.	**Intervention:** VR-based physical and cognitive training (60 min, 3/week, 12 weeks). **Control:** Combined physical (resistance, aerobic [50–75% heart rate] and balance exercises) and cognitive exercises.	(T1) at baseline; (T2) three months.	↑ Gait speed and stride length in ST and DT conditions in VR group** ↑ Gait speed and cadence only in ST in Control Group** No differences between groups*.

*MCI* Mild cognitive impairment, *RCT* Randomized Controlled Trial, *aMCI* Amnestic mild cognitive impairment, *MMSE* Mini-mental State Examination, *L3* Third lumbar vertebra level, *HR* Harmonic ratio that represent the smoothness of trunk movement, *VT* Vertical direction, *PCT* Combined simultaneous Physical and Cognitive Training, *PT* Physical Training, *CT* Cognitive Training, *ST* Single task, *DT* Dual task. WSC Walking Stroop Carpet test, *TUG* Timed Up an Go Test, *EO* Eyes open, *EC* Eyes closed, *CoM* Center of mass, *AP* Anterior-posterior, *ML* Medial-lateral, *MIP* Memory Intervention Program, *TTC* Taoist Tai Chi, *MoCA* Montreal Cognitive Assessment, *CTSIB* Clinical Test of Sensory Integration and Balance, *MVPA* Moderate-to-Vigorous Physical Activity, *VR* Virtual Reality

↑ Increased. ↓Decreased

*p > 0.05. **p < 0.05. ***p < 0.001

**Table 2:** Summary of included Cohort studies which compared Instrumented Objective Functional Parameters between Confirmed MCI and a Control Group

Study (first author and year)	Study Design	Study Characteristics (groups, number of participants, mean age)	MCI Diagnostic Criteria	Instrumented Functional Assessment	Instrument	Data Collection (follow-up)	Main results in MCI
Gillain et al. [63], 2015.	Pilot Cohort Study.	- MCI +: *n* = 9. 74.44 years old. - MCI -: *n* = 4. 70.00 years old.	Petersen Criteria [15]. CDR = 0.5 [46]. MMSE > 24 [49].	Walking at preferred speed (40 m) in ST and DT conditions.	Tri-axial accelerometric (Locometrix®) attached to the L3.	(T1) at baseline; (T2) one year; (T3) four years.	↑Gait speed in ST and in DT in MCI- than in MCI+ **. ↑Symmetry in DT in MCI- than in MCI+ **. ↓Gait performances in DT compared to ST.
Hayes et al. [64], 2008.	Transversal and longitudinal study (paired comparison and repeated measure ANOVA)	- Healthy Group: n = 7. 90 years old. - MCI: *n* = 7. 88.44 years old.	All: MMSE ≥24 [49]. Control: CDR = 0 [46]. MCI: CDR = 0.5 [46].	Activity in the home, amount of variance in activity, tracking visitors, absences from the home, and walking speed.	Motion sensors and magnetic contact sensors placed in home, and wireless contact switches.	(T1) mean of 315 days.	↑COV in the median gait speed in MCI compared with Healthy group **. ↑24-h wavelet variance in MCI Group than Healthy Group (↑variance in the day-to-day pattern of activity)**.
Ansai et al. [65]. 2018.	Longitudinal prospective study.	- AD: *n* = 37. 78.5 years old. - MCI: *n* = 38. 74.75 years old.	MCI Group: CDR = 0.5 [46]. MMSE > 24 [49]. Pfeffer [66].	TUG	Qualisys ProReflex motion analysis system with seven cameras.	(T1) at baseline; (T2) six months.	↓Total time to perform the TUG in MCI vs AD**. ↑ Gait speed in the walking forward subtask in MCI vs AD**. ↓ Time in the turn subtask in MCI vs AD**. ↑ Gait speed in the walking back subtask in MCI vs AD**. ↓ Time in the turn-to-sit subtask in MCI vs AD**.
Dodge et al. [67], 2012.	Longitudinal (Latent trajectory model). Part of cohort study.	- aMCI: *n* = 8. 84,5 years old. - naMCI: *n* = 31. 83.8 years old. - Healthy Group: *n* = 54. 84.9 years old.	ALL: CDR ≤ 0.5 [46]. MMSE > 24 [49]. MCI: Petersen Criteria [11].	Walking speed and its variability; total daily activity, visitors and time out of home.	Motion sensors and contact sensors fixed in the homes, and wireless contact switches.	(T1) at baseline; (T2) mean of 2.6 ± 1.0 years.	Slow gait speed in naMCI**. ↑or↓ baseline COV of gait speed groups in naMCI. ↓Gait speed in MCI than in Healthy Group**. ↑COV of gait speed in MCI than in Healthy Group**.
Pieruccini- Faria et al. [68], 2018.	Part of a prospective cohort study.	- MCI: *n* = 52. 73.7 years old. - Healthy Group: *n* = 27. 71.7 years old.	Control: - CDR = 0 [46]. - MoCA ≥27 [47]. MCI: - CDR = 0.5 [46]. - MoCA < 26 [47].	Walking speed in ST and DT conditions.	Electronic walkway (lenght: 6 m) embedded with sensors.	(T1) at baseline; (T2) two years; (T3) four years; (T4) five years.	↓ Gait speed in DT conditions in MCI**. ↓Step length adjustments in DT conditions in MCI**. ↓ Gait speed in MCI**.
Montero-Odasso et al. [69], 2009.	Reliability study.	- MCI: *n* = 11. 76.6 years old.	Petersen Criteria [14]. CDR = 0.5 [46]. MoCA < 26 [47]. MMSE > 24 [49].	Gait performance under ST and DT conditions.	Electronic walkway (GAITRite® System. Lenght: 6 m).	(T1) at baseline; (T2) one week.	↓Mean gait speed under DT conditions**. ↑Gait variability on stride time, step time, and double support time under DT conditions**.

*MCI* Mild cognitive impairment, *MCI +* MCI who will develop AD, *MCI* -MCI who will not develop AD, *CDR* Clinical Dementia Rating score, *MMSE* Mini-mental State Examination, *ST* Simple task, *DT* Dual task, *L3* Third lumbar vertebra level, *ANOVA* Analysis of Variance, *COV* Coefficient of variation, *AD* Alzheimer Disease, *TUG* Timed Up an Go Test, *aMCI* Amnestic mild cognitive impairment, *naMCI* Non-amnestic mild cognitive impairment, *MoCA* Montreal Cognitive Assessment, *GV* Gait velocity

↑Higher. ↓Lower

*p > 0.05. **p < 0.05. ***p < 0.001

**Table 3:** Summary of included Cohort studies examined the relationship between Kinematic Functional Parameters and an incident MCI

Study (first author and year)	Study Design	Study Characteristics (groups, number of participants, mean age)	MCI Diagnostic Criteria	Instrumented Functional Assessment	Instrument	Data Collection (follow-up)	Main results in MCI
Byun et al. [70], 2018.	Prospective cohort study.	Healty: *n* = 91. 67.3 years old.	Not diagnosis MCI at baseline: CDR = 0 [46]. MMSE > 24 [49]. Winblad Criteria [13] for diagnosis of MCI.	Walking at usual pace (20 m).	Tri-axial accelerometer (FITMETER®) at the level of the 3rd–4th lumbar vertebra.	(T1) at baseline; (T2) 2 years; (T3) median duration was 47.1 months.	↑Gait variability was a significant predictor of MCI (HR = 11.97, 95% CI = 1.29–111.37)***. Gait speed was slightly associated with incident MCI risk (HR = 5.04, 95% CI = 0.53–48.18) **.
Akl et al. [71], 2015.	Longitudinal study (trajectory with time window vector machines and random forests).	Older adults: *n* = 97. NS, 70 years old and + .	Cognitively Healthy: - CDR < 0.5 [46]. - MMSE > 24 [49]. MCI: - CDR = 0.5 [46]. - MMSE > 24 [49].	Walking speed and general activity in the home. Visitors and absences from the home.	Motion sensors and wireless contact switches placed in the home.	(T1) at baseline; (T2) one year; (T3) two years; (T4) three years.	Trajectories of weekly gait speed, COV of the gait speed, COV of the morning and evening gait speeds could detect MCI in older adults.
Akl et al. [72], 2015.	Longitudinal study (linear regression).	Older adults: *n* = 15. NS, 70 years old and + .	Cognitively Healthy: -CDR < 0.5 [46]. MCI: -CDR = 0.5 [46].	Walking speed in home.	Motion sensors on the ceiling in areas such as a hallway or a corridor.	(T1) at baseline; (T2) one year; (T3) two years; (T4) three years.	Gait speed distributions was different in the subjects when cognitively intact and when having MCI. Transitioning to MCI, daily activities were less distinguishable and often occurred later.
Buchman et al. [73], 2019.	Longitudinal cohort study.	Older adults: *n* = 1249. 80.0 years old.	MCI: - MMSE > 24 [49].	Walking at their self-selected Speed (10 m). TUG. Standing Posture with closed eyes.	Wearable sensor on the lower back.	(T1) at baseline; (T2) during 3.6 years.	↓ Cadence and regularity were associated with incident MCI **. Gait speed and gait variability were not associated with incident MCI *.

*MCI* Mild cognitive impairment, *CDR* Clinical Dementia Rating score, *MMSE* Mini-mental State Examination, *HR* Cox proportional Hazard, *CI* Confidence Interval, *NS* Not Specified, *COV* Coefficient of variation

↑Higher. ↓Decreased

*p > 0.05. **p < 0.05. ***p < 0.001

**Table 4:** Instruments used in kinematic analysis

Instrument	Papers n, %	References
Tri-axial accelerometer (e.g. Locometrix®, etc.)	4, 23.5%	[56, 60, 63, 70]
Electronic walkway (e.g. GAITRite®, etc.)	4, 23.5%	[57, 59, 68, 69]
Wearable sensors	2, 12%	[58, 73]
Digital Balance Platform	1, 6%	[59]
Inertial measurement units (IMUs)	1, 6%	[61]
Motion and contact sensors	4, 23.5%	[64, 67, 71, 72]
Qualisys ProReflex motion analysis System (cameras)	1, 6%	[65]
GAIT Up System.	1, 6%	[62]

**Table 5:** Criteria for MCI diagnosis reported in studies

Criteria	Papers n, %	References
Petersen et al. [11, 12, 14–16]	7, 41%	[56–59, 63, 67, 69]
Winblad et al. [13]	2, 12%	[60, 70]
CDR [41]	9, 53%	[63–65, 67–72]
MoCA [42]	5, 29%	[59, 61, 62, 68, 69]
MMSE [44]	11, 65%	[56, 59, 60, 63–65, 67, 69–71, 73]
Pfeiffer [60]	1, 6%	[65]

*MCI* Mild Cognitive Impairment, *CDR* Clinical Dementia Rating, *MoCA* Montreal Cognitive Assessment, *MMSE* Mini-Mental State Examination

### Functional objective parameters after physical activity interventions

RCTs showed that gait speed, cadence, stride length, smoothness of trunk movement in the vertical direction could be improved by aerobic exercises (60% of aged predicted maximal heart rate), especially when aerobic exercises are performed alongside cognitive stimulation exercises or others physical exercises such as muscle strength training, postural balance retraining, or gait training [56, 57, 62]. Stride time and the total time to perform the TUG also could be improved by the same interventions [56, 57]. The center of mass sway in anterior-posterior and medial-lateral directions also may be improved by the balance training [58].

### Functional objective parameters predicting MCI or discriminating MCI patients from a control group

Cohort studies suggested that a slower gait speed in ST condition and, above all, in DT conditions (counting backwards) was the parameter that best discriminated patients with MCI from cognitively healthy individuals [63, 67, 68]. Larger gait speed variability could also discriminate patients with MCI from cognitively healthy individuals [64, 67]. However, the total time to perform the TUG and the different subtask of the TUG, was the parameter which best discriminated patients with MCI from patients with Alzheimer disease instead of the gait speed, because patients with MCI took lower time in performing the TUG [65]. On the other hand, cohort studies showed that larger gait variability or larger gait speed variability could predict an incident MCI [70, 71]. Decreased cadence and walk-regularity also were associated with an incident MCI [73].

### Methodological quality

The methodological quality assessment of RCTs included is shown in supplementary Table 1. In summary, RCTs showed an unclear risk of bias, being the allocation concealment and the blinding of outcome assessment the ítems worst defined. Most of the included cohort studies (60%) reported low risk of bias while the rest (40%) showed moderate risk of bias (supplementary Table 2). Comparability was the main source of bias in cohort studies. The GRADE criteria showed a low level of evidence per outcome (supplementary Table 3).

## Discussion

### Statement of principal findings and comparision with others studies

The objective of this study was to review the current state of knowledge on the presence of functional kinematic parameters which may predict a future risk of MCI, could discriminate patients with MCI from a control group and could even be improved after different physical activity interventions. To our knowledge, this is the first systematic review that provides a comprehensive overview of longitudinal studies (RCTs and cohort studies) using an instrumented kinematic assessment of functional task as outcome measures or as parameters which could be impaired in patients with MCI or may predict an incident MCI. Furthermore, most of the studies included in this review were published after 2015, which indicates the novelty of the topic [57–63, 65, 68, 70–73].

Cohort studies showed that slower gait speed in ST condition and DT conditions (counting backwards), as well as a larger gait speed variability were the parameters that best discriminated patients with MCI from cognitively healthy individuals [64, 67, 68]. Slower gait speed in ST and DT conditions, as well as the time to perform the TUG were the parameters that best discriminated patients with MCI from patients with Alzheimer disease [63, 65]. Some studies have identified a slower gait speed in patients with MCI in comparison with cognitively healthy individuals [25–27, 29, 34, 35, 40]. Bahureksa et al. [40] also showed that a shorter stride length, a longer time to perform the stride (stride time) and a larger variability of these parameters may discriminate patients with MCI from cognitively healthy individuals. Verghese et al. [29] added a decreased cadence, a larger swing time variability, and a longer time to perform the swing phase, stance phase and double support phase, as parameters which could be impaired in patients with MCI in comparison with cognitively healthy controls. A previous systematic review also demonstrated slower gait speed and larger gait speed variability in patients with MCI than in cognitively healthy individuals [74].

Thus, larger gait variability, above all in DT conditions, could be associated with MCI [26]. In our review, cohort studies showed that a larger gait variability or a larger gait speed variability could predict an incident MCI [70, 71]. Several studies have also reported that a slow gait speed and a larger gait variability seem to be the main parameters which could predict a future cognitive decline and may be useful in the early detection of MCI [24, 26, 28, 30–33, 36, 75]. Decreased cadence, walk-regularity and slower gait speed were other kinematic parameters in our review which may be associated with an incident MCI [70, 73].

In the current review, RCTs suggested that gait speed, stride length, stride time, balance and the time to perform the TUG may be improved by aerobic exercises (60% of aged predicted maximal heart rate), especially when aerobic exercises are performed alongside cognitive stimulation exercises or others physical exercises such as muscle strength training, postural balance retraining, or gait training [56–58, 61]. Nevertheless, sample sizes were small in most of the included studies [56–59, 61, 62]. A previous study showed limited evidence on intervention effects on stride time variability [76] although this parameter seems to be a important predictor of MCI [58, 61, 63]. The combination of aerobic exercise, balance training and cognitive training could help reduce the risk of further cognitive impairment and may improve cognition, mobility, balance and quality of life [41, 43]. Furthermore, some systematic reviews and meta-analysis showed that aerobic and resistance (strength) exercises, join cognitive training could improve cognitive function, activities in daily living and mood [77–80].

### Strengths and weaknesses of the study

The strengths of this systematic review included the use of a pre-specified protocol registered on PROSPERO, the PRISMA checklist, the NOS and The Cochrane Collaboration’s tool to determine the risk of bias of included studies and the GRADE criteria to assess the level of evidence per outcome. Furthermore, this review only included studies which provided a validated diagnostic criteria of MCI. There are several limitations that should be mentioned. First, it is possible that some studies were not identified, although we conducted a robust search strategy in order to avoid it. Second, the lack of uniformity among the study design (e.g. walking distance, variables measured, different instruments used in kinematic analysis) should be taken into account when interpreting the results. In the literature, it has been demonstrated that participant walking strategy changes with walking distance, resulting in a significant effect on gait variability [81], so walking distance could be highly relevant in order to measure gait variability as a marker for MCI. Furthermore, studies did not report the reliability, validity or responsiveness of the instruments used in kinematic analysis, so we could not show what motion capture instrument is the most effective to perform the kinematic analysis. Third, RCTs reported an “unclear” risk of bias, so no firm conclusions should be drawn.

### Implications for clinical practice

Our results showed kinematic gait parameters which could be impaired and may predict an incident MCI. This is an important step forward in developing a clinically validated approach for measuring MCI related functional deficits which could predict a future risk of MCI and could even help its early diagnosis, although further studies are required in order to validate the findings of this review. Findings of this systematic review also could be useful for promoting specific interventions which could revert the functional changes associated with MCI, since RCTs included in this systematic review have demonstrated that physical activity interventions could improve some functional objective parameters.

### Implications for further research

Despite the promising results of the present study, some flaws observed in most of the included studies in this review should be resolved. Hence, there are some recommendations to guide future research: (i) studies should use the same instrument to perform the kinematic analysis which would allow a better comparison of data between studies; (ii) these instruments should be valid and reliable as established in the Cosmin taxonomy; (iii) RCTs and Cohort studies with high quality of evidence should be conducted since studies included in this systematic review often showed an unclear risk of bias and a low quality of evidence; (iv) Clinical trials which use functional objective parameters as outcome measures of physical activity interventions in MCI also should be conducted.

## Conclusion

Slower gait speed in ST condition and, above all, in DT conditions and larger gait speed variability are the parameters that best discriminate patients with MCI from a control group. Slower gait speed and larger gait variability may also predict an incident MCI or could even help its early diagnosis. Some functional objective parameters such as gait speed, stride length, stride time, balance and the time to perform the TUG may be improved by aerobic exercises when aerobic exercises are performed alongside cognitive stimulation exercises or others physical exercises such as muscle strength training, postural balance retraining, or gait training. Although most of the cohort studies showed a low risk of bias, RCTs reported unclear risk of bias and GRADE criteria showed a low level of evidence per outcome, so further studies are required to confirm our findings.

## Supplementary information

**Additional file 1 Supplementary Appendix A. PRISMA Checklist**. It includes the PRISMA checklist with the item reported in this trial.

**Additional file 2 Supplementary Appendix B. Search Strategy.** It includes the complete search strategy which was carried out with all search terms.

**Additional file 3 Supplementary Appendix C. Checklist for selection of studies.** It shows a checklist conducted to select studies which could be included in the present trial.

**Additional file 4 Supplementary Appendix D. Excluded studies in the second screening.** It contains references of excluded studies in the second screening since they did not meet inclusion criteria.

**Additional file 5 Supplementary Appendix E. Conflict of interest of included studies.** It shows the conflict of interests of included studies in the results.

**Additional file 6 Supplementary Table 1. Methodological Quality of included RCT (The Cochrane Collaboration’s tool).** It includes the risk of bias of RCTs included in the manuscript.

**Additional file 7 Supplementary Table 2. Methodological Quality of included prospective longitudinal studies (The Newcastle Ottawa Scale (NOS)).** It shows the risk of bias of included prospective non-randomized longitudinal studies.

**Additional file 8 Supplementary Table 3. Summary of findings and Quality of evidence assessment (GRADE).** It contains the quality of the evidence of each outcome informed at least by two studies with the same design based on the GRADE criteria.

## Data Availability

Not Applicable – this manuscript does not contain any data.

## Abbreviations

MCI: Mild Cognitive Impairment
AMED: The Allied and Complementary Medicine Database
CINAHL: Cumulative Index to Nursing and Allied Health Literature
PEDro: Physiotherapy Evidence Database
RCTs: Randomized Controlled Trials
DT: Dual Task conditions
ADL: Activities of Daily Living
IADL: Instrumental Activities of Daily Living
SPPB: Short Physical Performance Battery
TUG: Timed Up and Go Test
HGST: Hand Grip Strength Test
STS: Sit-To Stand Test
WST: Walking Speed ​​Test
ST: Single Task condition
NOS: Newcastle-Ottawa Quality Assessment Scale
GRADE: Grading of Recommendations Assessment,Development and Evaluation
PRISMA: Preferred Reporting Items for Systematic Reviews and Meta-Analyses
PROSPERO: International Prospective Register of Systematic Reviews
MeSH: Medical Subject Headings
CDR: Clinical Dementia Rating
MoCA: Montreal Cognitive Assessment
MMSE: Mini-Mental State Examination
