# Supplementary material for: Functional parameters indicative of mild cognitive impairment: a systematic review using instrumented kinematic assessment
Source: BMC Geriatr. 2020 Aug 10;20:282. doi: 10.1186/s12877-020-01678-6 (PMC7418187; doi:10.1186/s12877-020-01678-6)
Supplement: Supplementary file 2 — Additional file 2 Supplementary Appendix B. Search Strategy. It includes the complete search strategy which was carried out with all search terms. [file 12877_2020_1678_MOESM2_ESM.docx]

**Appendix A.** Search Strategy

1. PubMed, PEDro, Web Of Science, EMBASE, CINAHL, AMED: (Acceleromet*) OR kinematic) OR kinematic analysis) OR kinetics) AND six minute walk test) OR 6 minute walk test) AND mild cognitive impairment. 201.
2. PubMed, PEDro, Web Of Science, EMBASE, CINAHL, AMED: (Acceleromet*) OR kinematic) OR kinematic analysis) OR kinetics) AND timed up and go) OR TUG) AND mild cognitive impairment. 1054.
3. PubMed, PEDro, Web Of Science, EMBASE, CINAHL, AMED: (Acceleromet*) OR kinematic) OR kinematic analysis) OR kinetics) AND gait speed test) OR walking speed test) AND mild cognitive impairment. 701.
4. PubMed, PEDro, Web Of Science, EMBASE, CINAHL, AMED: (Acceleromet*) OR kinematic) OR kinematic analysis) OR kinetics) AND short performance physical battery) OR SPPB) AND mild cognitive impairment. 59.
5. PubMed, PEDro, Web Of Science, EMBASE, CINAHL, AMED: (Acceleromet*) OR kinematic) OR kinematic analysis) OR kinetics) AND sit to stand test) AND mild cognitive impairment. 55.
6. PubMed, PEDro, Web Of Science, EMBASE, CINAHL, AMED: (Acceleromet*) OR kinematic) OR kinematic analysis) OR kinetics) AND single leg stance test) OR one leg stance test) AND mild cognitive impairment. 18.
7. PubMed, PEDro, Web Of Science, EMBASE, CINAHL, AMED: (Acceleromet*) OR kinematic) OR kinematic analysis) OR kinetics) AND functional reach test) AND mild cognitive impairment. 22.
8. PubMed, PEDro, Web Of Science, EMBASE, CINAHL, AMED: (Acceleromet*) OR kinematic) OR kinematic analysis) OR kinetics) AND romberg test) AND mild cognitive impairment. 4.
9. PubMed, PEDro, Web Of Science, EMBASE, CINAHL, AMED: (Acceleromet*) OR kinematic) OR kinematic analysis) OR kinetics) AND functional task) AND mild cognitive impairment. 125.

**Grey Literature**

1. Open Grey, Grey Literature in Health Research, New York Academy of Medicine Grey Literature Report: Acceleromet* OR kinematic OR kinematic analysis OR kinetics AND six minute walk test OR 6 minute walk test OR six minute walk AND mild cognitive impairment. 0.
2. Open Grey, Grey Literature in Health Research, New York Academy of Medicine Grey Literature Report: Acceleromet* OR kinematic OR kinematic analysis OR kinetics AND timed up and go OR timed up and go test OR TUG AND mild cognitive impairment. 0.
3. Open Grey, Grey Literature in Health Research, New York Academy of Medicine Grey Literature Report: Acceleromet* OR kinematic OR kinematic analysis OR kinetics AND gait speed test OR walking speed test OR gait speed OR walking speed AND mild cognitive impairment. 0.
4. Open Grey, Grey Literature in Health Research, New York Academy of Medicine Grey Literature Report: Acceleromet* OR kinematic OR kinematic analysis OR kinetics AND short performance physical battery OR SPPB AND mild cognitive impairment. 0.
5. Open Grey, Grey Literature in Health Research, New York Academy of Medicine Grey Literature Report: Acceleromet* OR kinematic OR kinematic analysis OR kinetics AND sit to stand test OR sit to stand AND mild cognitive impairment. 0.
6. Open Grey, Grey Literature in Health Research, New York Academy of Medicine Grey Literature Report: Acceleromet* OR kinematic OR kinematic analysis OR kinetics AND single leg stance test OR one leg stance test OR single leg stance OR one leg stance AND mild cognitive impairment. 0.
7. Open Grey, Grey Literature in Health Research, New York Academy of Medicine Grey Literature Report: Acceleromet* OR kinematic OR kinematic analysis OR kinetics AND functional reach test OR functional reach AND mild cognitive impairment. 0.
8. Open Grey, Grey Literature in Health Research, New York Academy of Medicine Grey Literature Report: Acceleromet* OR kinematic OR kinematic analysis OR kinetics AND romberg test AND mild cognitive impairment. 0.
9. Open Grey, Grey Literature in Health Research, New York Academy of Medicine Grey Literature Report: Acceleromet* OR kinematic OR kinematic analysis OR kinetics AND functional task AND mild cognitive impairment. 0.
