# Supplementary material for: Functional parameters indicative of mild cognitive impairment: a systematic review using instrumented kinematic assessment
Source: BMC Geriatr. 2020 Aug 10;20:282. doi: 10.1186/s12877-020-01678-6 (PMC7418187; doi:10.1186/s12877-020-01678-6)
Supplement: Supplementary file 3 — Additional file 3 Supplementary Appendix C. Checklist for selection of studies. It shows a checklist conducted to select studies which could be included in the present trial. [file 12877_2020_1678_MOESM3_ESM.docx]

**Appendix B.** A short checklist for the selection of relevant studies based on inclusion and exclusion criteria.

| Item | Question | Action |
| --- | --- | --- |
| 1 | Was the study published as full-text paper? | Yes, go to the next question  No, exclude |
| 2 | Did the study include people with mild cognitive impairment? | Yes, go to the next question  No, exclude |
| 3 | Did the study report on the relationship between functional parameters and mild cognitive impairment during the performance of functional task? | Yes, go to the next question  No, exclude |
| 4 | Did the study include a kinematic analysis? | Yes, go to the next question  No, exclude |
| 5 | Did the study include a longitudinal prospective design? | Yes, go to the next question  No, exclude |
| 6 | Did the study use an objetive or validated methods to confirm the MCI diagnosis? | Yes, go to the next question  No, exclude |
| 7 | Was the study written in English or Spanish? | Yes, include study  No, exclude |
