# Supplementary material for: Functional parameters indicative of mild cognitive impairment: a systematic review using instrumented kinematic assessment
Source: BMC Geriatr. 2020 Aug 10;20:282. doi: 10.1186/s12877-020-01678-6 (PMC7418187; doi:10.1186/s12877-020-01678-6)
Supplement: Supplementary file 4 — Additional file 4 Supplementary Appendix D. Excluded studies in the second screening. It contains references of excluded studies in the second screening since they did not meet inclusion criteria. [file 12877_2020_1678_MOESM4_ESM.docx]

**Appendix C**. Excluded studies in the second screening (n= 260).

Not mild cognitive impairment. Other neurologic disease or older adults.

| 1 | Weiss A, Herman T, Plotnik M, Brozgol M, Giladi N, Hausdorff JM. An instrumented timed up and go: the added value of an accelerometer for identifying fall risk in idiopathic fallers. Physiol Meas. 2011;32(12): 2003–18. |
| --- | --- |
| 2 | Mirelman A, Patritti BL, Bonato P, Deutsch JE. Effects of virtual reality training on gait biomechanics of individuals post-stroke. Gait Posture. 2010;31(4): 433–7. |
| 3 | Leigh Hollands K, Hollands MA, Zietz D, Miles Wing A, Wright C, van Vliet P. Kinematics of Turning 180° During the Timed Up and Go in Stroke Survivors With and Without Falls History. Neurorehabil Neural Repair. 2010;24(4): 358–67. |
| 4 | McLoughlin JV, Barr CJ, Patritti B, Crotty M, Lord SR, Sturnieks DL. Fatigue induced changes to kinematic and kinetic gait parameters following six minutes of walking in people with multiple sclerosis. Disabil Rehabil. 2016;38(6): 535–43. |
| 5 | Beauchet O, Allali G, Montero-Odasso M, Sejdić E, Fantino B, Annweiler C. Motor phenotype of decline in cognitive performance among community-dwellers without dementia: Population-based study and meta-analysis. Brucki S, editor. PLoS One. 2014;9(6): e99318. doi: 10.1371/journal.pone.0099318 |
| 6 | Lauretani F, Galuppo L, Costantino C, Ticinesi A, Ceda G, Ruffini L, et al. Parkinson’s disease (PD) with dementia and falls is improved by AChEI? A preliminary study report. Aging Clin Exp Res. 2016;28(3): 551–5. |
| 7 | Bonnyaud C, Pradon D, Vaugier I, Vuillerme N, Bensmail D, Roche N. Timed Up and Go test: Comparison of kinematics between patients with chronic stroke and healthy subjects. Gait Posture. 2016;49: 258–63. |
| 8 | Saraiva N, Guimarães F, Lopes A, Papathanasiou J, Ferreira A. Feasibility of whole-body gait kinematics to assess the validity of the six-minute walk test over a 10-m walkway in the elderly. Biomed Signal Process Control. 2018;42: 202–9. |
| 9 | Yang F, Hassani A, Brost V, Mourey F, Kubicki A. Kinematic analysis of motor strategies in frail aged adults during the Timed Up and Go: how to spot the motor frailty? Clin Interv Aging. 2015;10: 505–513. doi: [10.2147/CIA.S74755](https://dx.doi.org/10.2147%2FCIA.S74755) |
| 10 | Olney SJ, Griffin MP, McBride ID. Temporal, Kinematic, and Kinetic Variables Related to Gait Speed in Subjects With Hemiplegia: A Regression Approach. Phys Ther. 1994;74(9): 872–85. |
| 11 | Straudi S, Manca M, Aiello E, Ferraresi G, Cavazza S, Basaglia N. Sagittal plane kinematic analysis of the six-minute walk test: a classification of hemiplegic gait. Eur J Phys Rehabil Med. 2009;45(3): 341–7. |
| 12 | Wagenaar RC, Beek WJ. Hemiplegic gait: A kinematic analysis using walking speed as a basis. J Biomech. 1992;25(9): 1007–15. |
| 13 | Wüest S, Massé F, Aminian K, Gonzenbach R, de Bruin ED. Reliability and validity of the inertial sensor-based Timed “Up and Go” test in individuals affected by stroke. J Rehabil Res Dev. 2016;53(5): 599–610. |
| 14 | Tyrell CM, Roos MA, Rudolph KS, Reisman DS. Influence of Systematic Increases in Treadmill Walking Speed on Gait Kinematics After Stroke. Phys Ther. 2011;91(3): 392-403. |
| 15 | Carlson RH, Huebner DR, Hoarty CA, Whittington J, Haynatzki G, Balas MC, et al. Treadmill gait speeds correlate with physical activity counts measured by cell phone accelerometers. Gait Posture. 2012;36(2): 241–8. |
| 16 | Hawkins KM, Sergio LE. Visuomotor impairments in older adults at increased Alzheimer’s disease risk. J Alzheimer’s Dis. 2014; 42(2):607–21. |
| 17 | Kim CM, Eng JJ. Magnitude and pattern of 3D kinematic and kinetic gait profiles in persons with stroke: relationship to walking speed. Gait Posture. 2004;20(2): 140–6. |
| 18 | Malatesta D, Canepa M, Menendez Fernandez A. The effect of treadmill and overground walking on preferred walking speed and gait kinematics in healthy, physically active older adults. Eur J Appl Physiol. 2017;117(9): 1833–43. |
| 19 | Campillay Guzmán J, Guzmán Silva R, Guzmán-Venegas R. Reproducibilidad de los tiempos de ejecución de la prueba de Timed Up and Go, medidos con acelerómetros de smartphones en personas mayores residentes en la comunidad. Rev Esp Geriatr Gerontol. 2017;52(5): 249–52. |
| 20 | Lord S, Rochester L, Hetherington V, Allcock LM, Burn D. Executive dysfunction and attention contribute to gait interference in ‘off’ state Parkinson’s Disease. Gait Posture. 2010;31(2): 169–74. |
| 21 | Ponti M, Bet P, Oliveira CL, Castro PC. Better than counting seconds: Identifying fallers among healthy elderly using fusion of accelerometer features and dual-task Timed Up and Go. Barkley J, editor. PLoS One. 2017;12(4): e0175559 |
| 22 | Weiss A, Herman T, Plotnik M, Brozgol M, Maidan I, Giladi N, et al. Can an accelerometer enhance the utility of the Timed Up and Go Test when evaluating patients with Parkinson’s disease? Med Eng Phys. 2010;32(2): 119–25. |
| 23 | de Souza e Silva EMG, Ribeiro TS, da Silva CC, Pereira Costa MF, Cavalcanti C, Rodrigues AR. Effects of constraint-induced movement therapy for lower limbs on measurements of functional mobility and postural balance in subjects with stroke: a randomized controlled trial. Top Stroke Rehabil. 2017;24(8): 555–61. |
| 24 | Zampieri C, Di Fabio RP. Balance and Eye Movement Training to Improve Gait in People With Progressive Supranuclear Palsy: Quasi-Randomized Clinical Trial. Phys Ther. 2008;88(12): 1460–73. |
| 25 | Zhuang J, Huang L, Wu Y, Zhang Y. The effectiveness of a combined exercise intervention on physical fitness factors related to falls in community-dwelling older adults. Clin Interv Aging. 2014;9: 131–140. |
| 26 | O. Bello, J.A. Sanchez, V. Lopez-Alonso, G. Márquez, L. Morenilla, X. Castro, et al. The effects of treadmill or overground walking training program on gait in Parkinson’s disease. Gait Posture. 2013;38(4): 590-595. |
| 27 | Hsieh Y, Liing R, Lin K, Wu C, Liou T, Lin J, et al. Sequencing bilateral robot-assisted arm therapy and constraint-induced therapy improves reach to press and trunk kinematics in patients with stroke. J Neuroeng Rehabil. 2016;13(1): 31. |
| 28 | Buckinx F, Beaudart C, Maquet D, Demonceau M, Crielaard JM, Reginster JY, et al. Evaluation of the impact of 6-month training by whole body vibration on the risk of falls among nursing home residents, observed over a 12-month period: a single blind, randomized controlled trial. Aging Clin Exp Res. 2014;26(4): 369–76. |
| 29 | Fielding RA, Guralnik JM, King AC, Pahor M, McDermott MM, Tudor-Locke C, et al. Dose of physical activity, physical functioning and disability risk in mobility-limited older adults: Results from the LIFE study randomized trial. PLoS One. 2017;12(8): e0182155. |
| 30 | Richards CL, Malouin F, Bravo G, Dumas F, Wood-Dauphinee S. The Role of Technology in Task-Oriented Training in Persons with Subacute Stroke: A Randomized Controlled Trial. Neurorehabil Neural Repair. 2004;18(4): 199–211. |
| 31 | Lin K, Huang P, Chen Y, Wu C, Huang W. Combining Afferent Stimulation and Mirror Therapy for Rehabilitating Motor Function, Motor Control, Ambulation, and Daily Functions After Stroke. Neurorehabil Neural Repair. 2014;28(2): 153–62. |
| 32 | Kleiner AFR, Pacifici I, Vagnini A, Camerota F, Celletti C, Stocchi F, et al. Timed Up and Go evaluation with wearable devices: Validation in Parkinson’s disease. J Bodyw Mov Ther. 2018;22(2): 390–5. |
| 33 | Sadowska D, Gumny M, Osinski W. Are the timed up and go test and functional reach test useful predictors of temporal and spatial gait parameters in elderly people? Hum Mov. 2016;17(3): 148–53. |
| 34 | Akahashi T, Ishida K, Hirose D, Nagano Y, Okumiya K, Nashinaga M, et al. Vertical ground reaction force shape is associated with gait parameters, timed up and go, and functional reach in elderly females. J Rehabil Med. 2004;36(1): 42–5. |
| 35 | Galán-Mercant A, Barón-López FJ, Labajos-Manzanares MT, Cuesta-Vargas AI. Reliability and criterion-related validity with a smartphone used in timed-up-and-go test. Biomed Eng Online. 2014;13: 156. |
| 36 | Rumble DD. Relationship Between Walking Speed and Kinematic Trajectory Complexity in People with Poststroke Hemiparesis. 2017;1-1. |
| 37 | Smith E, Walsh L, Doyle J, Greene B, Blake C. The reliability of the quantitative timed up and go test (QTUG) measured over five consecutive days under single and dual-task conditions in community dwelling older adults. Gait Posture. 2016;43: 239–44. |
| 38 | Zampieri C, Salarian A, Carlson-Kuhta P, Aminian K, Nutt JG, Horak FB. The instrumented timed up and go test: potential outcome measure for disease modifying therapies in Parkinson’s disease. J Neurol Neurosurg Psychiatry. 2010;81(2): 171–6. |
| 39 | Julie D. Ries, John L. Echternach LN, Michelle GB. Test-retest reliability and minimal detectable change scores for the Timed “Up & Go” Test, the Six-Minute Walk Test, and gait speed in people with Alzheimer disease. Phys Ther. 2009;89(6): 569–79. |
| 40 | Zampieri C, Salarian A, Carlson-Kuhta P, Aminian K, Nutt JG, Horak FB. Assessing mobility at home in people with early Parkinson’s disease using an instrumented Timed Up and Go test. Parkinsonism Relat Disord. 2011;17(4): 277–80. |
| 41 | Merchán-Baeza JA, González-Sánchez M, Cuesta-Vargas AI. Comparison of kinematic variables obtained by inertial sensors among stroke survivors and healthy older adults in the Functional Reach Test: cross-sectional study. Biomed Eng Online. 2015;14(1): 49. |
| 42 | Son M, Youm C, Cheon S, Kim J, Lee M, Kim Y, et al. Evaluation of the turning characteristics according to the severity of Parkinson disease during the timed up and go test. Aging Clin Exp Res. 2017;29(6): 1191–9. |
| 43 | Motl RW, Weikert M, Suh Y, Sosnoff JJ, Pula J, Soaz C, et al. Accuracy of the actibelt((R)) accelerometer for measuring walking speed in a controlled environment among persons with multiple sclerosis. Gait Posture. 2012;35(2): 192–6. |
| 44 | Doheny EP, Walsh C, Foran T, Greene BR, Fan CW, Cunningham C, et al. Falls classification using tri-axial accelerometers during the five-times-sit-to-stand test. Gait Posture. 2013;38(4): 1021–5. |
| 45 | López-Pascual J, Hurtado Abellán J, Inglés M, Espí-López G, Serra-Añó P. Reliability of variables measured with an Android device during a modified timed up and go test in patients with Alzheimer’s disease. Gait Posture. 2018;65: 484–5. |
| 46 | Perez-Cruzado D, Gonzalez-Sanchez M, Cuesta-Vargas AI. Differences in Kinematic Variables in Single-Leg Stance between Patients with Stroke and Healthy Elderly People Measured with Inertial Sensors: A Cross-Sectional Study. J Stroke Cerebrovasc Dis. 2018;27(1): 229–39. |
| 47 | Rochester L, Burn DJ, Woods G, Jon Godwin AN. Does auditory rhythmical cueing improve Gait in people with Parkinson’s disease and cognitive impairment? A feasibility study. Mov Disord. 2009;24(6): 839–45. |
| 48 | Mitsuru Yoneyama, Hiroshi Mitoma, Nobuo Sanjo, Maya Higuma, Hiroo Terashi, Takanori Yokota. Ambulatory Gait Behavior in Patients with Dementia: A Comparison with Parkinson’s Disease. IEEE Trans Neural Syst Rehabil Eng. 2016;24(8): 817–26. |
| 49 | James Yungjen Tung, Rhiannon Victoria Rose, Emnet Gammada, Isabel Lam, Eric Alexander Roy, Sandra EBP. Measuring life space in older adults with mild-to-moderate alzheimer’s disease using mobile phone GPS. Gerontology. 2014;60(2): 154–62. |
| 50 | Joe Verghese, Cuiling Wang, Richard B. Lipton, Roee Holtzer. Motoric cognitive risk syndrome and the risk of dementia. J Gerontol A Biol Sci Med Sci. 2013;68(4): 412–8. |
| 51 | Gorecka MM, Vasylenko O, Espenes J, Waterloo K, Rodríguez-Aranda C. The impact of age-related hearing loss and lateralized auditory attention on spatiotemporal parameters of gait during dual-tasking among community dwelling older adults. Exp Gerontol. 2018;111: 253–62. |
| 52 | Baston C, Mancini M, Schoneburg B, Horak F, Rocchi L. Postural strategies assessed with inertial sensors in healthy and parkinsonian subjects. Gait Posture. 2014;40(1): 70–5. |
| 53 | Morag E. Taylor, Kim Delbaere, A. Stefanie Mikolaizak, Stephen R. Lord, Jacqueline C.T. Close. Gait parameter risk factors for falls under simple and dual task conditions in cognitively impaired older people. Gait Posture. 2013;37(1): 126–30. |
| 54 | Olivier Beauchet, Cyrille P. Launay, Julia Chabot, Elise J. Levinoff, Gilles Allali. Subjective memory impairment and gait variability in cognitively healthy individuals: Results from a cross-sectional pilot study. J Alzheimer’s Dis. 2017;55(3): 965–71. |
| 55 | Ylva Cedervall, Kjartan Halvorsen, Anna Cristina Aberg. A longitudinal study of gait function and characteristics of gait disturbance in individuals with Alzheimer’s disease. Gait Posture. 2014;39(4): 1022–7. |
| 56 | Calazans Nogueira LA, dos Santos LT, Sabino PG, Papais Alvarenga RM, Santos Thuler LC. Walking execution is not affected by divided attention in patients with multiple sclerosis with no disability, but there is a motor planning impairment. Arq Neuropsiquiatr. 2013;71(8):521–6. |
| 57 | Lynne R. Sheffler, Paul N. Taylor, Stephanie Nogan Bailey, Douglas D. Gunzler, Jaap H. Buurke, Maarten J. IJzerman, et al. Surface peroneal nerve stimulation in lower limb hemiparesis: effect on quantitative gait parameters. Am J Phys Med Rehabil 2015 May  94(5): 341-357. |
| 58 | Bonnyaud C, Pradon D, Zory R, Bensmail D, Vuillerme N, Roche N. Does a single gait training session performed either overground or on a treadmill induce specific short-term effects on gait parameters in patients with hemiparesis? A randomized controlled study. Top Stroke Rehabil. 2013;20(6): 509-518. |
| 59 | Sofianidis G, Hatzitaki V, Douka S, Grouios G. Effect of a 10-week traditional dance program on static and dynamic balance control in elderly adults. J Aging Phys Act 2009;17(2): 167-180. |
| 60 | Thielman G. Insights into upper limb kinematics and trunk control one year after task-related training in chronic post-stroke individuals. J Hand Ther. 2013;26(2): 156–61. |
| 61 | Wu C, Trombly CA, Lin K, Tickle-Degnen L. A kinematic study of contextual effects on reaching performance in persons with and without stroke: Influences of object availability. Arch Phys Med Rehabil. 2000;81(1): 95–101. |
| 62 | Woodbury ML, Howland DR, McGuirk TE, Davis SB, Senesac CR, Kautz S, et al. Effects of Trunk Restraint Combined With Intensive Task Practice on Poststroke Upper Extremity Reach and Function: A Pilot Study. Neurorehabil Neural Repair. 2009;23(1): 78–91. |
| 63 | Summers JJ, Kagerer FA, Garry MI, Hiraga CY, Loftus A, Cauraugh JH. Bilateral and unilateral movement training on upper limb function in chronic stroke patients: A TMS study. J Neurol Sci. 2007;252(1): 76–82. |
| 64 | Lin K-C, Wu C-Y, Wei T-H, Gung C, Lee C-Y, Liu J-S. Effects of modified constraint-induced movement therapy on reach-to-grasp movements and functional performance after chronic stroke: a randomized controlled study. Clin Rehabil. 2007;21(12): 1075–86. |
| 65 | Lin K, Chen Y, Chen C, Wu C, Chang Y. The Effects of Bilateral Arm Training on Motor Control and Functional Performance in Chronic Stroke: A Randomized Controlled Study. Neurorehabil Neural Repair. 2010;24(1): 42–51. |
| 66 | Michaelsen SM, Dannenbaum R, Levin MF. Task-Specific Training With Trunk Restraint on Arm Recovery in Stroke. Stroke. 2006;37(1): 186–92. |
| 67 | Wu C, Lin K, Chen H, Chen I, Hong W. Effects of Modified Constraint-Induced Movement Therapy on Movement Kinematics and Daily Function in Patients With Stroke: A Kinematic Study of Motor Control Mechanisms. Neurorehabil Neural Repair. 2007;21(5): 460–6. |
| 68 | Braendvik SM, Koret T, Helbostad JL, Loras H, Brathen G, Hovdal HO, et al. Treadmill Training or Progressive Strength Training to Improve Walking in People with Multiple Sclerosis? A Randomized Parallel Group Trial. Physiother Res Int. 2016;21(4): 228–36. |
| 69 | Liao W, Wu C, Hsieh Y, Lin K, Chang W. Effects of robot-assisted upper limb rehabilitation on daily function and real-world arm activity in patients with chronic stroke: a randomized controlled trial. Clin Rehabil. 2012;26(2): 111–20. |
| 70 | Lucareli PR, Lima MO, Lima FPS, de Almeida JG, Brech GC, D’Andréa Greve JM. Gait analysis following treadmill training with body weight support versus conventional physical therapy: a prospective randomized controlled single blind study. Spinal Cord. 2011;49(9): 1001–7. |
| 71 | Arnold C, Lanovaz J, Oates A, Craven B, Butcher S. The Effect of Adding Core Stability Training to a Standard Balance Exercise Program on Sit to Stand Performance in Older Adults: A Pilot Study. J Aging Phys Act. 2015;23(1): 95–102. |
| 72 | Shahraki M, Sohrabi M, Taheri Torbati HR, Nikkhah K, NaeimiKia M. Effect of rhythmic auditory stimulation on gait kinematic parameters of patients with multiple sclerosis. J Med Life. 2017;10(1): 33-37. |
| 73 | Ribeiro T, Britto H, Oliveira D, Silva E, Galvão E, Lindquist A. Effects of treadmill training with partial body weight support and the proprioceptive neuromuscular facilitation method on hemiparetic gait: a randomized controlled study. Eur J Phys Rehabil Med. 2013;49(4): 451-461. |
| 74 | Williams G, Clark R, Schache A, Fini NA, Moore L, Morris ME, McCrory PR. Training conditions influence walking kinematics and self-selected walking speed in patients with neurological impairments. J Neurotrauma. 2011;28(2): 281-287. |
| 75 | Persch LN, Ugrinowitsch C, Pereira G, Rodacki AL. Strength training improves fall-related gait kinematics in the elderly: a randomized controlled trial. Clin Biomech. 2009;24(10): 819-825. |
| 76 | Nooijen CF1, Ter Hoeve N, Field-Fote EC. Gait quality is improved by locomotor training in individuals with SCI regardless of training approach. J Neuro Engineering Rehabil. 2009;6: 36.doi: 10.1186/1743-0003-6-36. |
| 77 | Davies BL, Arpin DJ, Liu M, Reelfs H, Volkman KG, Healey K, et al. Two different types of high-frequency physical therapy promote improvements in the balance and mobility of persons with multiple sclerosis. Arch Phys Med Rehabil. 2016;97(12): 2095-2101. |
| 78 | Byl N, Zhang W, Coo S, Tomizuka M. Clinical impact of gait training enhanced with visual kinematic biofeedback: Patients with Parkinson’s disease and patients stable post stroke. Neuropsychologia. 2015;79: 332–43. |
| 79 | Gimmon Y, Riemer R, Kurz I, Shapiro A, Debbi R, Melzer I. Perturbation exercises during treadmill walking improve pelvic and trunk motion in older adults—A randomized control trial. Arch Gerontol Geriatr. 2018;75: 132–8. |
| 80 | Lewek MD, Cruz TH, Moore JL, Roth HR, Dhaher YY, Hornby TG. Allowing Intralimb Kinematic Variability During Locomotor Training Poststroke Improves Kinematic Consistency: A Subgroup Analysis From a Randomized Clinical Trial. Phys Ther. 2009;89(8): 829–39. |
| 81 | Brasileiro A, Gama G, Trigueiro L, Ribeiro T, Silva E, Galvão É, et al. Influence of visual and auditory biofeedback on partial body weight support treadmill training of individuals with chronic hemiparesis: a randomized controlled clinical trial. Eur J Phys Rehabil Med. 2015;51(1): 49-58. |
| 82 | Mahtani GB, Kinnaird CR, Connolly M, Holleran CL, Hennessy PW, Woodward J, et al. Altered sagittal- and frontal-plane kinematics following high-intensity stepping training versus conventional interventions in subacute stroke. Phys Ther. 2017;97(3): 320-329. |
| 83 | Cheng J-S, Yang Y-R, Cheng S-J, Lin P-Y, Wang R-Y. Effects of Combining Electric Stimulation With Active Ankle Dorsiflexion While Standing on a Rocker Board: A Pilot Study for Subjects With Spastic Foot After Stroke. Arch Phys Med Rehabil. 2010;91(4): 505–12. |
| 84 | El-Tamawy M, Darwish M, Khallaf M. Effects of augmented proprioceptive cues on the parameters of gait of individuals with Parkinson′s disease. Ann Indian Acad Neurol. 2012;15(4): 267. |
| 85 | Okubo Y, Menant J, Udyavar M, Brodie MA, Barry BK, Lord SR, et al. Transfer effects of step training on stepping performance in untrained directions in older adults: A randomized controlled trial. Gait Posture. 2017;54: 50–5. |
| 86 | Pau M, Corona F, Coghe G, Marongiu E, Loi A, Crisafulli A, et al. Quantitative assessment of the effects of 6 months of adapted physical activity on gait in people with multiple sclerosis: a randomized controlled trial. Disabil Rehabil. 2018;40(2): 144–51. |
| 87 | Paoloni M, Mangone M, Scettri P, Procaccianti R, Cometa A, Santilli V. Segmental Muscle Vibration Improves Walking in Chronic Stroke Patients With Foot Drop: A Randomized Controlled Trial. Neurorehabil Neural Repair. 2010;24(3): 254–62. |
| 88 | Van Swearingen JM, Perera S, Brach JS, Wert D, Studenski SA. Impact of Exercise to Improve Gait Efficiency on Activity and Participation in Older Adults With Mobility Limitations: A Randomized Controlled Trial. Phys Ther. 2011;91(12): 1740–51. |
| 89 | Jaywant A, Ellis TD, Roy S, Lin C-C, Neargarder S, Cronin-Golomb A. Randomized Controlled Trial of a Home-Based Action Observation Intervention to Improve Walking in Parkinson Disease. Arch Phys Med Rehabil. 2016;97(5): 665–73. |
| 90 | Givon N, Zeilig G, Weingarden H, Rand D. Video-games used in a group setting is feasible and effective to improve indicators of physical activity in individuals with chronic stroke: a randomized controlled trial. Clin Rehabil. 2016;30(4): 383–92. |
| 91 | Mansfield A, Wong JS, Bryce J, Brunton K, Inness EL, Knorr S, et al. Use of Accelerometer-Based Feedback of Walking Activity for Appraising Progress With Walking-Related Goals in Inpatient Stroke Rehabilitation. Neurorehabil Neural Repair. 2015;29(9): 847–57. |
| 92 | Kim J-S, Oh D-W, Kim S-Y, Choi J-D. Visual and kinesthetic locomotor imagery training integrated with auditory step rhythm for walking performance of patients with chronic stroke. Clin Rehabil. 2011;25(2): 134–45. |
| 93 | Straudi S, Benedetti MG, Venturini E, Manca M, Foti C, Basaglia N. Does robot-assisted gait training ameliorate gait abnormalities in multiple sclerosis? A pilot randomized-control trial. NeuroRehabilitation. 2013;33(4): 555-563. |
| 94 | Azadian E, Torbati HRT, Kakhki ARS, Farahpour N. The effect of dual task and executive training on pattern of gait in older adults with balance impairment: A Randomized controlled trial. Arch Gerontol Geriatr. 2016;62: 83–9. |
| 95 | Cirstea CM, Ptito A, Levin MF. Feedback and Cognition in Arm Motor Skill Reacquisition After Stroke. Stroke. 2006;37(5): 1237–42. |
| 96 | Wu C, Yang C, Chuang L, Lin K, Chen H, Chen M, et al. Effect of Therapist-Based Versus Robot-Assisted Bilateral Arm Training on Motor Control, Functional Performance, and Quality of Life After Chronic Stroke: A Clinical Trial. Phys Ther. 2012;92(8): 1006–16. |
| 97 | Batchelor FA, Williams SB, Wijeratne T, Said CM, Petty S. Balance and Gait Impairment in Transient Ischemic Attack and Minor Stroke. J Stroke Cerebrovasc Dis. 2015;24(10): 2291–7. |
| 98 | Weiss A, Mirelman A, Giladi N, Barnes LL, Bennett DA, Buchman AS, et al. Transition Between the Timed up and Go Turn to Sit Subtasks: Is Timing Everything? J Am Med Dir Assoc. 2016;17(9): 864.e9-864.e15. |
| 99 | Yu F, Bilberg A, Dalgas U, Stenager E. Fatigued patients with multiple sclerosis can be discriminated from healthy controls by the recordings of a newly developed measurement system (FAMOS): a pilot study. Disabil Rehabil Assist Technol. 2013;8(1): 77–83. |
| 100 | Barha CK, Hsiung G-YR, Best JR, Davis JC, Eng JJ, Jacova C, et al. Sex Difference in Aerobic Exercise Efficacy to Improve Cognition in Older Adults with Vascular Cognitive Impairment: Secondary Analysis of a Randomized Controlled Trial. Burns J, editor. J Alzheimers Dis. 2017;60(4): 1397–410. |
| 101 | De Cock A-M, Fransen E, Perkisas S, Verhoeven V, Beauchet O, Vandewoude M, et al. Comprehensive quantitative spatiotemporal gait analysis identifies gait characteristics for early dementia subtyping in community dwelling older adults. Front Neurol. 2019;10(APR). |
| 102 | Finsterwalder S, Wuehr M, Gesierich B, Dietze A, Konieczny M-J, Schmidt R, et al. Minor gait impairment despite white matter damage in pure small vessel disease. Ann Clin Transl Neurol. 2019;6(10): 2026–36. |
| 103 | Stuart S, Parrington L, Morris R, Martini D-N, Fino P-C, King L-A. Gait measurement in chronic mild traumatic brain injury: A model approach. Hum Mov Sci. 2020;69. |
| 104 | Liparoti M, Della Corte M, Rucco R, Sorrentino P, Sparaco M, Capuano R, et al. Gait abnormalities in minimally disabled people with Multiple Sclerosis: A 3D-motion analysis study. Mult Scler Relat Disord. 2019;29: 100–7. |
| 105 | Purcell NL, Goldman JG, Ouyang B, Liu Y, Bernard B, O’Keefe JA. The effects of dual-task cognitive interference on gait and turning in Huntington’s disease. PLoS One. 2020;15(1): e0226827–e0226827. |
| 106 | Beerse M, Lelko M, Wu J. Biomechanical analysis of the timed up-and-go (TUG) test in children with and without Down syndrome. Gait Posture. 2019;68: 409–14. |
| 107 | Manor B, Greenstein PE, Davila-Perez P, Wakefield S, Zhou J, Pascual-Leone A. Repetitive Transcranial Magnetic Stimulation in Spinocerebellar Ataxia: A Pilot Randomized Controlled Trial. Front Neurol. 2019;10. |

Not Kinematic analysis.

| 1 | Dawson N, Judge KS, Gerhart H. Improved Functional Performance in Individuals With Dementia After a Moderate-Intensity Home-Based Exercise Program. J Geriatr Phys Ther. 2019;42(1): 18–27. | |  |  |
| --- | --- | --- | --- | --- |
| 2 | Persad C, Jones J, Ashton-Miller J, Alexander N, Giordani B. Executive function and gait in older adults with cognitive impairment. J Gerontol A Biol Sci Med Sci. 2008;63(12): 1350–5. | |  |  |
| 3 | Nielsen N, Simonsen A, Siersma V, Hasselbalch S, Hoegh P. The Diagnostic and Prognostic Value of a Dual-Tasking Paradigm in a Memory Clinic. Sergio L, editor. J Alzheimer’s Dis. 2018;61(3): 1189–99. | |  |  |
| 4 | Lamb S, Mistry D, Alleyne S, Atherton N, Brown D, Copsey B, et al. Aerobic and strength training exercise programme for cognitive impairment in people with mild to moderate dementia: the DAPA RCT. Health Technol Assess (Rockv). 2018;22(28): 1–201. | |  |  |
| 5 | Varela S, Ayán C, Cancela JM, Martín V. Effects of two different intensities of aerobic exercise on elderly people with mild cognitive impairment: a randomized pilot study. Clin Rehabil. 2012;26(5): 442–50. | |  |  |
| 6 | Rolenz E, Reneker J. Validity of the 8-Foot Up and Go, Timed Up and Go, and Activities-Specific Balance Confidence Scale in older adults with and without cognitive impairment. J Rehabil Res Dev. 2016;53(4): 511–8. | |  |  |
| 7 | Borges S, Radanovic M, Forlenza O. Correlation between functional mobility and cognitive performance in older adults with cognitive impairment. Aging, Neuropsychol Cogn. 2018;25(1): 23–32. | | |  |
| 8 | | Eggermont LH, Gavett BE, Volkers KM, Blankevoort CG, Scherder EJ, Jefferson AL, et al. Lower-Extremity Function in Cognitively Healthy Aging, Mild Cognitive Impairment, and Alzheimer’s Disease. Arch Phys Med Rehabil. 2010;91(4): 584–8. | | |
| 9 | | De Melo Borges S, Radanovic M, Forlenza OV. Functional Mobility in a Divided Attention Task in Older Adults With Cognitive Impairment. J Mot Behav. 2015;47(5): 378–85. | | |
| 10 | | Rüdiger S, Stuckenschneider T, Vogt T, Abeln V, Lawlor B, Olde Rikkert M, et al. Cognitive Impairment Is Reflected by an Increased Difference between Real and Imagined Timed Up and Go Test Performance. Dement Geriatr Cogn Disord. 2017;44(1–2): 55–62. | | |
| 11 | | Kovács E, Sztruhár Jónásné I, Karóczi CK, Korpos A, Gondos T. Effects of a multimodal exercise program on balance, functional mobility and fall risk in older adults with cognitive impairment: a randomized controlled single-blind study. Eur J Phys Rehabil Med. 2013;49(5): 639–48. | | |
| 12 | | Hagovská M, Olekszyová Z. Impact of the combination of cognitive and balance training on gait, fear and risk of falling and quality of life in seniors with mild cognitive impairment. Geriatr Gerontol Int. 2016;16(9): 1043–50. | | |
| 13 | | Casas-Herrero A, Cadore EL, Zambom-Ferraresi F, Idoate F, Millor N, Martínez-Ramirez A, et al. Functional capacity, muscle fat infiltration, power output, and cognitive impairment in institutionalized frail oldest old. Rejuvenation Res. 2013;16(5): 396–403. | | |
| 14 | | Metzger FG, Hobert MA, Ehlis A-C, Hasmann SE, Hahn T, Eschweiler GW, et al. Dual tasking for the differentiation between depression and mild cognitive impairment. Front Aging Neurosci. 2016;8: 235. | | |
| 15 | | Hooghiemstra AM, Ramakers IHGB, Sistermans N, Pijnenburg YAL, Aalten P, Hamel REG, et al. Gait Speed and Grip Strength Reflect Cognitive Impairment and Are Modestly Related to Incident Cognitive Decline in Memory Clinic Patients With Subjective Cognitive Decline and Mild Cognitive Impairment: Findings From the 4C Study. Journals Gerontol Ser A. 2017;72(6): 846–54. | | |
| 16 | | Knapstad MK, Steihaug OM, Aaslund MK, Nakling A, Naterstad IF, Fladby T, et al. Reduced Walking Speed in Subjective and Mild Cognitive Impairment. J Geriatr Phys Ther. 2017;00: 1-7. | | |
| 17 | | Beauchet O, Launay CP, Sejdić E, Allali G, Annweiler C. Motor imagery of gait: A new way to detect mild cognitive impairment? J Neuroeng Rehabil. 2014;11(1): 66. | | |
| 18 | | Pettersson AF, Olsson E, Wahlund. Effect of divided attention on gait in subjects with and without cognitive impairment. J Geriatr Psychiatry Neurol. 2007;20(1): 58–62. | | |
| 19 | | Tseng BY, Cullum CM, Zhang R. Older adults with amnestic mild cognitive impairment exhibit exacerbated gait slowing under dual-task challenges. Curr Alzheimer Res. 2014;11(5): 494–500. | | |
| 20 | | Doi T, Shimada H, Makizako H, Tsutsumimoto K, Uemura K, Anan Y, et al. Cognitive function and gait speed under normal and dual-task walking among older adults with mild cognitive impairment. BMC Neurol. 2014 ;14(1): 67. | | |
| 21 | | Veronese N, Stubbs B, Trevisan C, Bolzetta F, De Rui M, Solmi M, et al. What physical performance measures predict incident cognitive decline among intact older adults? A 4.4 year follow up study. Exp Gerontol. 2016;81: 110–8. | | |
| 22 | | McGough EL, Kelly VE, Logsdon RG, McCurry SM, Cochrane BB, Engel JM, et al. Associations between physical performance and executive function in older adults with mild cognitive impairment: gait speed and the timed “up and go” test. Phys Ther. 2011;91(8): 1198–207. | | |
| 23 | | Fujisawa C, Umegaki H, Okamoto K, Nakashima H, Kuzuya M, Toba K, et al. Physical Function Differences Between the Stages From Normal Cognition to Moderate Alzheimer Disease. J Am Med Dir Assoc. 2017;18(4): 368.e9-368.e15. | | |
| 24 | | Uemura K, Shimada H, Makizako H, Doi T, Yoshida D, Tsutsumimoto K, et al. Cognitive function affects trainability for physical performance in exercise intervention among older adults with mild cognitive impairment. Clin Interv Aging. 2013;8: 97–102. | | |
| 25 | | Jiaojiao L, Mingyun S, Leichao L, Yi Xiaoyu P, Yu L. Effects of momentum-based dumbbell training on cognitive function in older adults with mild cognitive impairment: a pilot randomized controlled trial. Clin Interv Aging. 2015;11: 9-16. | | |
| 26 | | Choi W, Lee S. Ground Kayak Paddling Exercise Improves Postural Balance, Muscle Performance, and Cognitive Function in Older Adults with Mild Cognitive Impairment: A Randomized Controlled Trial. Med Sci Monit. 2018;24: 3909–15. | | |
| 27 | | Morag ET, Kim D, Stephen RL, A. Stefanie M, Henry B, Jacqueline CTC. Neuropsychological, physical, and functional mobility measures associated with falls in cognitively impaired older adults. J Gerontol A Biol Sci Med Sci. 2014;69(8): 987–95. | | |
| 28 | | G. Wadley V, Okonkwo O, Crowe M, Ross-Meadows L. Mild cognitive impairment and everyday function: Evidence of reduced speed in performing instrumental activities of daily living. Am J Geriatr Psychiatry. 2008;16(5): 416–24. | | |
| 29 | | Franssen EH, Souren LE, Torossian CL, Reisberg B. Equilibrium and limb coordination in mild cognitive impairment and mild Alzheimer’s disease. J Am Geriatr Soc. 1999;47(4): 463–9. | | |
| 30 | | Montero-Odasso M, Bergman H, Phillips N, Wong C, Sourial N, Chertkow H. Dual-tasking and gait in people with mild cognitive impairment. the effect of working memory. BMC Geriatr. 2009;9(1): 41. doi:10.1186/1471-2318-9-41 | | |
| 31 | | Buracchio T, Dodge HH, Howieson D, Wasserman D, Kaye J.The trajectory of gait speed preceding mild cognitive impairment. Arch Neurol. 2010;67(8): 980–6. | | |
| 32 | | [Makizako H](https://www.ncbi.nlm.nih.gov/pubmed/?term=Makizako%20H%5BAuthor%5D&cauthor=true&cauthor_uid=23915144), [Shimada H](https://www.ncbi.nlm.nih.gov/pubmed/?term=Shimada%20H%5BAuthor%5D&cauthor=true&cauthor_uid=23915144), [Doi T](https://www.ncbi.nlm.nih.gov/pubmed/?term=Doi%20T%5BAuthor%5D&cauthor=true&cauthor_uid=23915144), [Park H](https://www.ncbi.nlm.nih.gov/pubmed/?term=Park%20H%5BAuthor%5D&cauthor=true&cauthor_uid=23915144), [Yoshida D](https://www.ncbi.nlm.nih.gov/pubmed/?term=Yoshida%20D%5BAuthor%5D&cauthor=true&cauthor_uid=23915144), [Uemura K](https://www.ncbi.nlm.nih.gov/pubmed/?term=Uemura%20K%5BAuthor%5D&cauthor=true&cauthor_uid=23915144), et al. Poor balance and lower gray matter volume predict falls in older adults with mild cognitive impairment. BMC Neurol. 2013;13: 102. doi: 10.1186/1471-2377-13-102. | | |
| 33 | | Rosso AL, Verghese J, Metti AL, Boudreau RM, Aizenstein HJ, Kritchevsky S, et al. Slowing gait and risk for cognitive impairment. Neurology. 2017;89(4): 336–42.  doi: 10.1212/WNL.0000000000004153 | | |
| 34 | | Schepker CA, Leveille SG, Pedersen MM, Ward RE, Kurlinski LA, Grande L, et al. Effect of Pain and Mild Cognitive Impairment on Mobility. J Am Geriatr Soc. 2016;64(1): 138–43. doi: 10.1111/jgs.13869. | | |
| 35 | | Chong MS, Tay L, Chan M, Shiong Lim W, Ye R, King Tan E, et al. Prospective longitudinal study of frailty transitions in a community-dwelling cohort of older adults with cognitive impairment. BMC Geriatr. 2015;15: 175. doi: [10.1186/s12877-015-0174-1](https://dx.doi.org/10.1186%2Fs12877-015-0174-1) | | |
| 36 | | Kubicki A, Fautrelle L, Bourrelier J, Rouaud O, Mourey F.The early indicators of functional decrease in mild cognitive impairment. Front Aging Neurosci. 2016;8: 193. | | |
| 37 | | Pedersen MM, Holt NE, Grande L, Kurlinski LA, Beauchamp MK, Kiely DK, et al. Mild cognitive impairment status and mobility performance: an analysis from the Boston RISE study. J Gerontol A Biol Sci Med Sci. 2014;69(12): 1511–8. doi: 10.1093/gerona/glu063. | | |
| 38 | | Johansson H, Lundin-Olsson L, Littbrand H, Gustafson Y, Rosendahl E, Toots A. Cognitive function and walking velocity in people with dementia a comparison of backward and forward walking. Gait Posture. 2017;58: 481–6. | | |
| 39 | | Montero-Odasso M, Muir SW, Speechley M. Dual-task complexity affects gait in people with mild cognitive impairment: The interplay between gait variability, dual tasking, and risk of falls. Arch Phys Med Rehabil. 2012;93(2): 293–9. doi: 10.1016/j.apmr.2011.08.026. | | |
| 40 | | Díaz-Pelegrina A, Cabrera-Martos I, López-Torres I, Rodríguez-Torres J, Valenza MC. Effects of cognitive state on balance disturbances and gait disorders in institutionalised elderly. Rev Esp Geriatr Gerontol. 2016;51(2): 88–91. | | |
| 41 | | Makizako H, Doi T, Shimada H, Yoshida D, Tsutsumimoto K, Uemura K, et al. Does a multicomponent exercise program improve dual-task performance in amnestic mild cognitive impairment? A randomized controlled trial. Aging Clin Exp Res. 2012;24(6): 640–6. | | |
| 42 | | Boyle PA, Wilson RS, Buchman AS, Aggarwal NT, Tang Y, Arvanitakis Z, et al. Lower extremity motor function and disability in mild cognitive impairment. Exp Aging Res. 2007;33(3): 355–71. | | |
| 43 | | MacAulay RK, Wagner MT, Szeles D, Milano NJ. Improving Sensitivity to Detect Mild Cognitive Impairment: Cognitive Load Dual-Task Gait Speed Assessment. J Int Neuropsychol Soc. 2017;23(6): 493–501. doi: 10.1017/S1355617717000261. | | |
| 44 | | Doi T, Shimada H, Makizako H, Tsutsumimoto K, Hotta R, Nakakubo S, et al. Mild Cognitive Impairment, Slow Gait, and Risk of Disability: A Prospective Study. J Am Med Dir Assoc. 2015;16(12): 1082–6. doi: 10.1016/j.jamda.2015.07.007. | | |
| 45 | | Doi T, Makizako H, Tsutsumimoto K, Hotta R, Nakakubo S, Makino K, et al. Combined effects of mild cognitive impairment and slow gait on risk of dementia. Exp Gerontol. 2018;110: 146–50. | | |
| 46 | | Gonçalves J, Ansai JH, Masse FAA, Vale FAC, Takahashi ACM, Andrade LP. Dual-task as a predictor of falls in older people with mild cognitive impairment and mild Alzheimer’s disease: a prospective cohort study. Brazilian J Phys Ther. 2018;22(5): 417–23. | | |
| 47 | | Law LLF, Barnett F, Yau MK, Gray MA. Effects of functional tasks exercise on older adults with cognitive impairment at risk of Alzheimer’s disease: a randomised controlled trial. Age Ageing. 2014;43(6): 813–20. | | |
| 48 | | Hagovská M, Olekszyová Z. Relationships between balance control and cognitive functions, gait speed, and activities of daily living. Z Gerontol Geriatr. 2016;49(5): 379–85. | | |
| 49 | | Naidu AS, Vasudev A, Burhan AM, Ionson E, Montero-Odasso M. Does Dual-Task Gait Differ in those with Late-Life Depression versus Mild Cognitive Impairment? Am J Geriatr Psychiatry. 2019;27(1): 62-72. doi: 10.1016/j.jagp.2018.10.011 | | |
| 50 | | Rajtar-Zembaty A, Sałakowski A, Rajtar-Zembaty J, Starowicz-Filip A, Skalska A. Slow gait as a motor marker of mild cognitive impairment? the relationships between functional mobility and mild cognitive impairment. Aging, Neuropsychol Cogn. 2018;1–10. | | |
| 51 | | Kim S. Exploring the field application of combined cognitive-motor program with mild cognitive impairment elderly patients. J Exerc Rehabil. 2018;14(5): 817–20. | | |
| 52 | | Langoni C da S, Resende T de L, Barcellos AB, Cecchele B, da Rosa JN, Knob MS, et al. The effect of group exercises on balance, mobility, and depressive symptoms in older adults with mild cognitive impairment: a randomized controlled trial. Clin Rehabil. 2018: 269215518815218. | | |
| 53 | | Prohaska TR, Eisenstein AR, Satariano WA, Hunter R, Bayles CM, Kurtovich E, et al. Walking and the preservation of cognitive function in older populations. Gerontologist. 2009;49 Suppl 1(S1): S86-93. | | |
| 54 | | Boyle PA, Buchman AS, Wilson RS, Leurgans SE, Bennett DA. Physical frailty is associated with incident mild cognitive impairment in community-based older persons. J Am Geriatr Soc. 2010;58(2): 248–55. | | |
| 55 | | Nakatsuka M, Nakamura K, Hamanosono R, Takahashi Y, Kasai M, Sato Y, et al. A Cluster Randomized Controlled Trial of Nonpharmacological Interventions for Old-Old Subjects with a Clinical Dementia Rating of 0.5: The Kurihara Project. Dement Geriatr Cogn Dis Extra. 2015;5(2): 221–32. | | |
| 56 | | Lee SH, Han JH, Jin YY, Lee IH, Hong HR, Kang HS. Poor physical fitness is independently associated with mild cognitive impairment in elderly Koreans. Biol Sport. 2016;33(1): 57–62. | | |
| 57 | | Ansai JH, de Andrade LP, Masse FAA, Gonçalves J, de Medeiros Takahashi AC, Vale FAC, et al. Risk Factors for Falls in Older Adults With Mild Cognitive Impairment and Mild Alzheimer Disease. J Geriatr Phys Ther. 2017. doi: 10.1519/JPT.0000000000000135. | | |
| 58 | | Ansai JH, Andrade LP de, Rossi PG, Almeida ML, Carvalho Vale FA, Rebelatto JR. Association Between Gait and Dual Task With Cognitive Domains in Older People With Cognitive Impairment. J Mot Behav. 2018;50(4): 409–15. doi: 10.1080/00222895.2017.1363702. | | |
| 59 | | Langoni C da S, Resende T de L, Barcellos AB, Cecchele B, Knob MS, Silva T do N, et al. Effect of Exercise on Cognition, Conditioning, Muscle Endurance, and Balance in Older Adults With Mild Cognitive Impairment: A Randomized Controlled Trial. J Geriatr Phys Ther. 2018.  doi: 10.1519/JPT.0000000000000191. | | |
| 60 | | Cox K-L, Cyarto E-V, Ellis K-A, Ames D, Desmond P, Phal P, et al. A Randomized Controlled Trial of Adherence to a 24-Month Home-Based Physical Activity Program and the Health Benefits for Older Adults at Risk of Alzheimer’s Disease: The AIBL Active-Study. J Alzheimer’s Dis. 2019;70(s1): S187–205. | | |
| 61 | | Rajtar-Zembaty A, Rajtar-Zembaty J, Sałkowski A, Starowicz-Filip A, Skalska A. Global cognitive functioning and physical mobility in older adults with and without mild cognitive impairment: evidence and implications. Folia Med Cracov. 2019;59(1): 75–88. | | |
| 62 | | Yu L, Boyle P-A, Leurgans S-E, Wilson R-S, Bennett D-A, Buchman A-S. Incident Mobility Disability, Mild Cognitive Impairment, and Mortality in Community-Dwelling Older Adults. Neuroepidemiology. 2019;53(1–2): 55–62. | | |
| 63 | | Hoogendijk E-O, J. Rijnhart J-M, Skoog J, Robitaille A, van den Hout A, Ferrucci L, et al. Gait speed as predictor of transition into cognitive impairment: Findings from three longitudinal studies on aging. Exp Gerontol. 2020;129: 1-17. | | |
| 64 | | Kume Y, Takahashi T, Itakura Y, Lee S, Makizako H, Ono T, et al. Characteristics of Mild Cognitive Impairment in Northern Japanese Community-Dwellers from the ORANGE Registry. J Clin Med. 2019;8(11):1937. | | |
| 65 | | Lowe D-A, MacAulay R-K, Szeles D-M, Milano N-J, Wagner M-T. Dual-Task Gait Assessment in a Clinical Sample: Implications for Improved Detection of Mild Cognitive Impairment. J Gerontol B Psychol Sci Soc Sci. 2019;1: 1-26. | | |
| 66 | | Rosso A-L, Metti A-L, Faulkner K, Redfern M, Yaffe K, Launer L, et al. Complex Walking Tasks and Risk for Cognitive Decline in High Functioning Older Adults. J Alzheimer’s Dis [Internet]. 2019;71(s1): 65–73. | | |
| 67 | | Cullen S, Borrie M, Carroll S, Sarquis-Adamson Y, Pieruccini-Faria F, McKay S, et al. Are Cognitive Subtypes Associated with Dual-Task Gait Performance in a Clinical Setting? J Alzheimer’s Dis. 2019;71(s1): 57–64. | | |
| 68 | | Yoon B, Choi SH, Jeong JH, Park KW, Kim E-J, Hwang J, et al. Balance and Mobility Performance Along the Alzheimer’s Disease Spectrum. J Alzheimers Dis. 2020;73(2): 633–44. | | |
| 69 | | Harper KJ, Riley V, Petta A, Jacques A, Spendier N, Ingram K. Occupational therapist use of the “Timed Up and Go” test in a Memory Clinic to compare performance between cognitive diagnoses and screen for falls risk. Aust Occup Ther J. 2020;67(1): 13–21. | | |
| 70 | | Tian Q, Resnick S-M, Studenski S-A, Ferrucci L. Lap Time Variability From a 400-m Walk Is Associated With Future Mild Cognitive Impairment and Alzheimer’s Disease. J Am Med Dir Assoc. 2019;20(12): 1535-1539. | | |

Relationship between functional kinematic parameters and brain structures.

| 1 | Makizako H, Shimada H, Doi T, Yoshida D, Ito K, Kato T, et al. The association between decline in physical functioning and atrophy of medial temporal areas in community-dwelling older adults with amnestic and nonamnestic mild cognitive impairment. Arch Phys Med Rehabil. 2011;92(12): 1992–9. |
| --- | --- |
| 2 | Halloway S, Arfanakis K, Wilbur J, Schoeny ME, Pressler SJ. Accelerometer Physical Activity is Associated with Greater Gray Matter Volumes in Older Adults Without Dementia or Mild Cognitive Impairment. Journals Gerontol Ser B. 2018. doi: 10.1093/geronb/gby010. |
| 3 | Annweiler C, Beauchet O, Bartha R, Wells JL, Borrie MJ, Hachinski V, et al. Motor cortex and gait in mild cognitive impairment: A magnetic resonance spectroscopy and volumetric imaging study. Brain. 2013;136(3): 859–71. |
| 4 | Crockett RA, Hsu CL, Best JR, Liu-Ambrose T. Resting state default mode network connectivity, dual task performance, gait speed, and postural sway in older adults with mild cognitive impairment. Front Aging Neurosci. 2017;9: 423. doi: 10.3389/fnagi.2017.00423. |
| 5 | Onen F, Henry-Feugeas MC, Roy C, Baron G, Ravaud P. Mobility decline of unknown origin in mild cognitive impairment: An MRI-based clinical study of the pathogenesis. Brain Res. 2008;1222: 79–86. doi: 10.1016/j.brainres.2008.05.027 |
| 6 | Kose Y, Ikenaga M, Yamada Y, Morimura K, Takeda N, Ouma S, et al. Timed Up and Go test, atrophy of medial temporal areas and cognitive functions in community-dwelling older adults with normal cognition and mild cognitive impairment. Exp Gerontol. 2016;85: 81–7. |
| 7 | Beauchet O, Launay CP, Sekhon H, Montembeault M, Allali G. Association of hippocampal volume with gait variability in pre-dementia and dementia stages of Alzheimer disease: Results from a cross-sectional study. Exp Gerontol. 2019;115: 55–61. |
| 8 | Beauchet O, Launay CP, Annweiler C, Allali G. Hippocampal volume, early cognitive decline and gait variability: Which association? Exp Gerontol. 2015;61: 98–104. |
| 9 | Annweiler C, Beauchet O, Bartha R, Montero-Odasso M. Slow gait in MCI is associated with ventricular enlargement: Results from the Gait and Brain Study. J Neural Transm. 2013;120(7): 1083–92. |
| 10 | Allali G, Annweiler C, Predovan D, Bherer L, Beauchet O. Brain volume changes in gait control in patients with mild cognitive impairment compared to cognitively healthy individuals GAIT study results. Exp Gerontol. 2016;76: 72–9. |
| 11 | Doi T, Makizako H, Shimada H, Park H, Tsutsumimoto K, Uemura K, et al. Brain activation during dual-task walking and executive function among older adults with mild cognitive impairment: A fNIRS study. Aging Clin Exp Res. 2013;25(5): 539–44. |
| 12 | Sakurai R, Bartha R, Montero-Odasso M. Entorhinal Cortex Volume Is Associated With Dual-Task Gait Cost Among Older Adults With MCI: Results From the Gait and Brain Study. J Gerontol A Biol Sci Med Sci. 2019;74(5): 698–704. |
| 13 | Cosentino E, Palmer K, Della Pietà C, Mitolo M, Meneghello F, Levedianos G, et al. Association between Gait, Cognition, and Gray Matter Volumes in Mild Cognitive Impairment and Healthy Controls. Alzheimer Dis Assoc Disord. 2020. |
| 14 | Snir J-A, Bartha R, Montero-Odasso M. White matter integrity is associated with gait impairment and falls in mild cognitive impairment. Results from the gait and brain study. NeuroImage Clin. 2019;24. |
| 15 | [Halloway](https://pubmed.ncbi.nlm.nih.gov/?term=Halloway+S&cauthor_id=29432610) S, [Arfanakis](https://pubmed.ncbi.nlm.nih.gov/?term=Arfanakis+K&cauthor_id=29432610) K, [Wilbur](https://pubmed.ncbi.nlm.nih.gov/?term=Wilbur+J&cauthor_id=29432610) J, [Schoeny](https://pubmed.ncbi.nlm.nih.gov/?term=Schoeny+ME&cauthor_id=29432610) M-E, [Pressler](https://pubmed.ncbi.nlm.nih.gov/?term=Pressler+SJ&cauthor_id=29432610) S-J. Accelerometer Physical Activity is Associated with Greater Gray Matter Volumes in Older Adults Without Dementia or Mild Cognitive Impairment. 2020. |
| 16 | Allali G, Montembeault M, Saj A, Wong CH, Cooper-Brown LA, Bherer L, et al. Structural Brain Volume Covariance Associated with Gait Speed in Patients with Amnestic and Non-Amnestic Mild Cognitive Impairment: A Double Dissociation. J Alzheimer’s Dis. 2019;71: S29–39. |
| 17 | Beauchet O, Montembeault M, Barden J-M, Szturm T, Bherer L, Liu-Ambrose T, et al. Brain gray matter volume associations with gait speed and related structural covariance networks in cognitively healthy individuals and in patients with mild cognitive impairment: A cross-sectional study. Exp Gerontol. 2019;122: 116–22. |

Other Kinematic parameters different from functional parameters.

| 1 | Schröter A, Mergl R, Bürger K, Hampel H, Möller H-J, Hegerl U. Kinematic Analysis of Handwriting Movements in Patients with Alzheimer’s Disease, Mild Cognitive Impairment, Depression and Healthy Subjects. Dement Geriatr Cogn Disord. 2003;15(3): 132–42. |
| --- | --- |
| 2 | Garre-Olmo J, Faúndez-Zanuy M, López-de-Ipiña K, Calvó-Perxas L, Turró-Garriga O. Kinematic and Pressure Features of Handwriting and Drawing: Preliminary Results Between Patients with Mild Cognitive Impairment, Alzheimer Disease and Healthy Controls. Curr Alzheimer Res. 2017;14(9): 960-968. |
| 3 | Yu N-Y, Chang S-H. Kinematic Analyses of Graphomotor Functions in Individuals with Alzheimer’s Disease and Amnestic Mild Cognitive Impairment. J Med Biol Eng. 2016;36(3): 334–43. |
| 4 | Werner P, Rosenblum S, Bar-On G, Heinik J, Korczyn A. Handwriting process variables discriminating mild Alzheimer’s disease and mild cognitive impairment. Journals Gerontol - Ser B Psychol Sci Soc Sci. 2006;61(4): 228–36. |
| 5 | Toosizadeh N, Ehsani H, Wendel C, Zamrini E, Connor KO, Mohler J. Screening older adults for amnestic mild cognitive impairment and early-stage Alzheimer’s disease using upper-extremity dual-tasking. Sci Rep. 2019;9(1): 10911. |

Not instrumented analysis.

| 1 | Hauer K, Schwenk M, Zieschang T, Essig M, Becker C, Oster P. Physical Training Improves Motor Performance in People with Dementia: A Randomized Controlled Trial. J Am Geriatr Soc. 2012;60(1): 8–15. |
| --- | --- |
| 2 | Ansai JH, Andrade LP, Rossi PG, Takahashi ACM, Vale FAC, Rebelatto JR, et al. Gait, dual task and history of falls in elderly with preserved cognition, mild cognitive impairment, and mild Alzheimer’s disease. Brazilian J Phys Ther. 2017;21(2): 144–51. |

Not longitudinal prospective design.

| 1 | Bahureksa L, Najafi B, Saleh A, Sabbagh M, Coon D, Mohler MJ, et al. The Impact of Mild Cognitive Impairment on Gait and Balance: A Systematic Review and Meta-Analysis of Studies Using Instrumented Assessment. Gerontology. 2016;63(1): 67–83. |
| --- | --- |
| 2 | Lussier M, Lavoie M, Giroux S, Consel C, Guay M, Macoir J, et al. Early detection of mild cognitive impairment with in-home monitoring technologies using functional measures: A systematic review. IEEE J Biomed Heal Informatics. 2018: 1-1. |
| 3 | Ambrose AF, Noone ML, Pradeep VG, Johnson B, Salam KA, Verghese J. Gait and cognition in older adults: Insights from the Bronx and Kerala. Ann Indian Acad Neurol. 2010;13(SUPPL. 2): S99–103. |
| 4 | Allali G, Verghese J. Management of Gait Changes and Fall Risk in MCI and Dementia. Curr Treat Options Neurol. 2017;19(9): 29. |
| 5 | Martínez-Ramírez A, Martinikorena I, Lecumberri P, Gómez M, Millor N, Casas-Herrero A, et al. Dual Task Gait Performance in Frail Individuals with and without Mild Cognitive Impairment. Dement Geriatr Cogn Disord. 2016;42(1–2): 7–16. |
| 6 | Kikkert LHJ, Vuillerme N, van Campen JP, Appels BA, Hortobágyi T, Lamoth CJC. Gait characteristics and their discriminative power in geriatric patients with and without cognitive impairment. J Neuroeng Rehabil. 2017;14(1): 84 |
| 7 | Gillain S, Warzee E, Lekeu F, Wojtasik V, Maquet D, Croisier J-L, et al. The value of instrumental gait analysis in elderly healthy, MCI or Alzheimer’s disease subjects and a comparison with other clinical tests used in single and dual-task conditions. Ann Phys Rehabil Med. 2009;52(6): 453–74. |
| 8 | Deschamps T, Beauchet O, Annweiler C, Cornu C, Mignardot JB. Postural control and cognitive decline in older adults: Position versus velocity implicit motor strategy. Gait Posture. 2014;39(1): 628–30. |
| 9 | Nima Toosizadeh, Bijan Najafi, Eric M. Reiman, Reine M. Mager, Jaimeson K. Veldhuizen, Kathy O’Connor, et al. Upper-extremity dual-task function: An innovative method to assess cognitive impairment in older adults. Front Aging Neurosci. 2016;8: 167. |
| 10 | Bertoli M, Cereatti A, Trojaniello D, Avanzino L, Pelosin E, Del Din S, et al. Estimation of spatio-temporal parameters of gait from magneto-inertial measurement units: Multicenter validation among Parkinson, mildly cognitively impaired and healthy older adults. Biomed Eng Online. 2018;17(1): 58. |
| 11 | Seo K, Kim J, Oh DH, Ryu H, Choi H. Virtual daily living test to screen for mild cognitive impairment using kinematic movement analysis. Chen K, editor. PLoS One. 2017;12(7): e0181883. |
| 12 | König A, Klaming L, Pijl M, Demeurraux A, David R, Robert P. Objective measurement of gait parameters in healthy and cognitively impaired elderly using the dual-task paradigm. Aging Clin Exp Res. 2017;29(6): 1181–9. |
| 13 | De Cock AM, Perkisas S, Verhoeven V, Vandewoude M, Fransen E, Remmen R. The impact of cognitive impairment on the physical ageing process. Aging Clin Exp Res. 2018;30(11): 1297-1306. |
| 14 | Tanigawa T, Takechi H, Arai H, Yamada M, Nishiguchi S, Aoyama T. Effect of physical activity on memory function in older adults with mild Alzheimer’s disease and mild cognitive impairment. Geriatr Gerontol Int. 2014;14(4): 758–62. |
| 15 | Mignardot J-B, Beauchet O, Annweiler C, Cornu C, Deschamps T. Postural Sway, Falls, and Cognitive Status: A Cross-Sectional Study among Older Adults. J Alzheimer’s Dis. 2014;41(2): 431–9. |
| 16 | Ansai JH, Andrade LP de, Nakagawa TH, Vale FAC, Caetano MJD, Lord SR, et al. Cognitive Correlates of Timed Up and Go Subtasks in Older People With Preserved Cognition, Mild Cognitive Impairment, and Alzheimer’s Disease. Am J Phys Med Rehabil. 2017;96(10): 700–5. |
| 17 | Callisaya ML, Launay CP, Srikanth VK, Verghese J, Allali G, Beauchet O. Cognitive status, fast walking speed and walking speed reserve—the Gait and Alzheimer Interactions Tracking (GAIT) study. GeroScience. 2017;39(2): 231–9. |
| 18 | D. Maquet, F. Lekeu, E. Warzee, S. Gillain, V. Wojtasik, E. Salmon JP and JLC. Gait analysis in elderly adult patients with mild cognitive impairment and patients with mild Alzheimer’s disease: Simple versus dual task: A preliminary report. Clin Physiol Funct Imaging. 2010;30(1): 51–6. |
| 19 | Muir SW, Speechley M, Wells J, Borrie M, Gopaul K, Montero-Odasso M. Gait assessment in mild cognitive impairment and Alzheimer’s disease: The effect of dual-task challenges across the cognitive spectrum. Gait Posture. 2012;35(1): 96–100. |
| 20 | Perrochon A, Kemoun G, Watelain E, Dugué B, Berthoz A. The “Stroop Walking Task”: An innovative dual-task for the early detection of executive function impairment. Neurophysiol Clin. 2015;45(3): 181–90. |
| 21 | De Cock A-M, Fransen E, Perkisas S, Verhoeven V, Beauchet O, Remmen R, et al. Gait characteristics under different walking conditions: Association with the presence of cognitive impairment in community-dwelling older people. Gard SA, editor. PLoS One. 2017;12(6): e0178566. |
| 22 | Mirelman A, Weiss A, Buchman AS, Bennett DA, Giladi N, Hausdorff JM. Association between performance on timed up and go subtasks and mild cognitive impairment: Further insights into the links between cognitive and motor function. J Am Geriatr Soc. 2014;62(4): 673–8. |
| 23 | Ansai JH, de Andrade LP, Rossi PG, Nakagawa TH, Vale FAC, Rebelatto JR. Differences in Timed Up and Go Subtasks Between Older People With Mild Cognitive Impairment and Mild Alzheimer’s Disease. Motor Control. 2018;1–12. |
| 24 | Perrochon A, Kemoun G. The walking trail-making test is an early detection tool for mild cognitive impairment. Clin Interv Aging. 2014;9: 111–9. |
| 25 | König A, Crispim-Junior CF, Covella AGU, Bremond F, Derreumaux A, Bensadoun G, et al. Ecological assessment of autonomy in instrumental activities of daily living in dementia patients by the means of an automatic video monitoring system. Front Aging Neurosci. 2015;7: 98. |
| 26 | Allali G, Annweiler C, Blumen HM, Callisaya ML, De Cock AM, Kressig RW, et al. Gait phenotype from mild cognitive impairment to moderate dementia: Results from the GOOD initiative. Eur J Neurol. 2016;23(3): 527–41. |
| 27 | Nishiguchi S, Yorozu A, Adachi D, Takahashi M, Aoyama T. Association between mild cognitive impairment and trajectory-based spatial parameters during timed up and go test using a laser range sensor. J Neuroeng Rehabil. 2017;14(1): 78. |
| 28 | Beauchet O, Allali G, Launay C, Herrmann FR, Annweiler C. Gait variability at fast-pace walking speed: A biomarker of mild cognitive impairment? J Nutr Health Aging. 2013;17(3): 235–9. |
| 29 | Beauchet O, Blumen HM, Callisaya ML, De Cock AM, Kressig RW, Srikanth V, et al. Spatiotemporal Gait Characteristics Associated with Cognitive Impairment: A Multicenter Cross-Sectional Study, the Intercontinental “Gait, Cognition & Decline” Initiative. Curr Alzheimer Res. 2018;15(3): 273–82. |
| 30 | Allali G, Launay CP, Blumen HM, Callisaya ML, De Cock A-M, Kressig RW, et al. Falls, Cognitive Impairment, and Gait Performance: Results From the GOOD Initiative. J Am Med Dir Assoc. 2017;18(4): 335–40. |
| 31 | Montero-Odasso M, Oteng-Amoako A, Speechley M, Gopaul K, Beauchet O, Annweiler C, et al. The motor signature of mild cognitive impairment: results from the gait and brain study. J Gerontol A Biol Sci Med Sci. 2014;69(11): 1415–21. |
| 32 | de Oliveira Silva F, Ferreira JV, Plácido J, Chagas D, Praxedes J, Guimarães C, et al. Stages of mild cognitive impairment and Alzheimer’s disease can be differentiated by declines in timed up and go test: A systematic review and meta-analysis. Arch Gerontol Geriatr. 2019;85: 103941. |
| 33 | de Oliveira Silva F, Ferreira JV, Plácido J, Chagas D, Praxedes J, Guimarães C, et al. Gait analysis with videogrammetry can differentiate healthy elderly, mild cognitive impairment, and Alzheimer’s disease: A cross-sectional study. Exp Gerontol. 2020;131: 110816 |
| 34 | Cohen J-A, Verghese J. Gait and dementia. Handb Clin Neurol. 2019;167: 419–27 |
| 35 | Gorenko J-A, Smith A-P, Hundza S-R, Halliday D. W-R, DeCarlo C-A, Sheets D-J, et al. A socially-engaged lifestyle moderates the association between gait velocity and cognitive impairment. Aging Ment Health. 2020;1–9. |
| 36 | Choi J, Park J, Lee BI, Shin KJ, Yoo S, Kim H, et al. The Correlation between Cognition Screening Scores and Gait Status from Three-Dimensional Gait Analysis. J Clin Neurol. 2019;15(2): 152–8 |
| 37 | Xie H, Wang Y, Tao S, Huang S, Zhang C, Lv Z. Wearable Sensor-Based Daily Life Walking Assessment of Gait for Distinguishing Individuals With Amnestic Mild Cognitive Impairment. Front Aging Neurosci. 2019;11. |
| 38 | Schaat S, Koldrack P, Yordanova K, Kirste T, Teipel S. Real-Time Detection of Spatial Disorientation in Persons with Mild Cognitive Impairment and Dementia. Gerontology. 2020;66(1): 85–94 |
| 39 | Grande G, Triolo F, Nuara A, Welmer A-K, Fratiglioni L, Vetrano D-L. Measuring gait speed to better identify prodromal dementia. Exp Gerontol. 2019;124. |
| 40 | Sekhon H, Launay C-P, Chabot J, Allali G, Beauchet O. Motoric cognitive risk syndrome: Could it be defined through increased five-times-sit-to-stand test time, rather than slow walking speed? Front Aging Neurosci. 2019;11. |

Not validated mild cognitive impairment diagnosis of score os MMSE < 24.

| 1 | Werner C, Wiloth S, Lemke NC, Kronbach F, Jansen C-P, Oster P, et al. People with Dementia Can Learn Compensatory Movement Maneuvers for the Sit-to-Stand Task: A Randomized Controlled Trial. Fleiner T, editor. J Alzheimer’s Dis. 2017;60(1): 107–20. |
| --- | --- |
| 2 | Hauer K, Ullrich P, Dutzi I, Beurskens R, Kern S, Bauer J, et al. Effects of Standardized Home Training in Patients with Cognitive Impairment following Geriatric Rehabilitation: A Randomized Controlled Pilot Study. Gerontology. 2017;63(6): 495–506. |
| 3 | Werner C, Moustris GP, Tzafestas CS, Hauer K. User-Oriented Evaluation of a Robotic Rollator That Provides Navigation Assistance in Frail Older Adults with and without Cognitive Impairment. Gerontology. 2018;64(3): 278–90. |
| 4 | Taylor ME, Lasschuit DA, Lord SR, Delbaere K, Kurrle SE, Mikolaizak AS, et al. Slow gait speed is associated with executive function decline in older people with mild to moderate dementia: A one year longitudinal study. Arch Gerontol Geriatr. 2017;73: 148–53. |
| 5 | Harada K, Lee S, Lee S, Bae S, Harada K, Shimada H. Changes in objectively measured outdoor time and physical, psychological, and cognitive function among older adults with cognitive impairments. Arch Gerontol Geriatr. 2018;78: 190–5. |
| 6 | Auvinet B, Touzard C, Montestruc F, Delafond A, Goeb V. Gait disorders in the elderly and dual task gait analysis: a new approach for identifying motor phenotypes. J Neuroeng Rehabil. 2017;14(1): 1–14. |
| 7 | Montero-Odasso MM, Barnes B, Speechley M, Muir Hunter SW, Doherty TJ, Duque G, et al. Disentangling Cognitive-Frailty: Results From the Gait and Brain Study. J Gerontol A Biol Sci Med Sci. 2016;71(11):1476–82. |

Not incident MCI or Control Group.

| 1 | Pieruccini-Faria F, Sarquis-Adamson Y, Anton-Rodrigo I, Noguerón-García A, Bray N-W, Camicioli R, et al. Mapping Associations Between Gait Decline and Fall Risk in Mild Cognitive Impairment. J Am Geriatr Soc. 2019. |
| --- | --- |

Not Spanish or English language.

| 1 | Koskas P, Saad S, Belqadi S, Drunat O. Analyse clinique au cours d’une marche simple et avec une double tâche chez une population de sujets âgés ambulatoires consultant en gériatrie. Rev Neurol. 2010;166(3): 321–7. |
| --- | --- |
| 2 | Vanmairis J. L’imagerie motrice dans la rééducation de la marche des amputés trans-tibiaux d’origine vasculaire. Kinésithérapie, la Rev. 2018;18(194): 2–12. |
| 3 | Bi CF, Peng LJ, Mao LL, Du W, Liu L, Qian HR. Diagnostic value of gait speed and “Timed Up and Go” on Alzheimer’s disease. Chinese J Contemp Neurol Neurosurg. 2015;15(7): 565–9. |
| 4 | Faria C. Timed Up and Go Test für Schlaganfallpatienten. Z Physiother Krankengymnastik. 2010;62(8): 20–1. |
| 5 | Botolfsen P. Reliabilitet av den norske versjonen av Timed Up and Go (TUG). Fysioterapeuten. 2010;77(5): 16–7. |
| 6 | Tekeşin A, Tunç A, Güngen BD, Avci N, Bakış M, Perk Ş. A pulmonalis fizioterápia és az aerob edzésprogram javítja a kognitív funkciót és a fizikai terhelhetőséget. Ideggyogy Sz. 2018;71(11–12): 423–30. |
| 7 | Jamour M, Becker C, Synofzik M, Maetzler W. Gangveränderungen als Frühindikator einer Demenz. Z Gerontol Geriatr. 2012;45(1): 40–4. |
| 8 | Geelen RH, Hendrix LA, van Rooijen SM, Beurskens AJ, Koke AJ. Rubriek Meten in de praktijk. Timed Up and Go test. Ned Tijdschr Fysiother. 2007;117(3): 27–8. |
| 9 | Menezes AV, Aguiar A da S de, Alves EF, Quadros LB de, Bezerra PP. Efetividade de uma intervenção fisioterapêutica cognitivo-motora em idosos institucionalizados com comprometimento cognitivo leve e demência leve. Cien Saude Colet. 2016;21(11): 3459–67. |
| 10 | Schniepp R, Wuehr M, Schöberl F, Zwergal A. Erfassung motorisch-kognitiver Interaktionen bei Demenzerkrankungen im klinischen Alltag. Fortschritte der Neurol · Psychiatr. 2016;84(08): 469–79. |
| 11 | Parthum A, Kishek AA. Die Simulation seniorentypischer Einschränkungen beeinflusst den “timed-up-go-Test” (TUG). Pflegewissenschaft. 2016;18: 246–50. |
