# Supplementary material for: Functional parameters indicative of mild cognitive impairment: a systematic review using instrumented kinematic assessment
Source: BMC Geriatr. 2020 Aug 10;20:282. doi: 10.1186/s12877-020-01678-6 (PMC7418187; doi:10.1186/s12877-020-01678-6)
Supplement: Supplementary file 5 — Additional file 5 Supplementary Appendix E. Conflict of interest of included studies. It shows the conflict of interests of included studies in the results. [file 12877_2020_1678_MOESM5_ESM.docx]

**Appendix D.** Conflict of interest of the included studies.

| **Fist Author and Year** | **Conflict of Interest** |
| --- | --- |
| Doi et al [56], 2013. | No commercial party had a direct financial interest in the  results of the research supporting the article had or confered a benefit on the authors or on any organization with which the authors are associated. |
| Donnezan et al [57], 2018. | Not reported. |
| Schwenk et al [58], 2016. | No conflict of interest. |
| Fogarty et al [59], 2016. | Not reported. |
| Bae et al [60], 2018. | No conflict of interest. |
| Delbroek et al [61], 2017. | Not reported. |
| Liao et al [62], 2019. | Not reported. |
| Gillain et al [63], 2015. | None of the authors had a financial or personal relationship with people or organizations that could inappropriately  influence that work. |
| Hayes et al [64], 2008. | Not reported. |
| Ansai et al [65], 2018. | Not reported. |
| Dodge et al [67], 2012. | No conflict of interest. |
| Pieruccini- Faria et al [68], 2018. | Not reported. |
| Montero-Odasso et al [69], 2009. | No conflict of interest. |
| Byun et al [70], 2018. | No conflict of interest. |
| Akl et al [71], 2015. | Not reported. |
| Akl et al [72], 2015. | Not reported. |
| Buchman et al [73], 2019. | The authors did not declare any conflict of interest. |
