# Supplementary material for: Functional parameters indicative of mild cognitive impairment: a systematic review using instrumented kinematic assessment
Source: BMC Geriatr. 2020 Aug 10;20:282. doi: 10.1186/s12877-020-01678-6 (PMC7418187; doi:10.1186/s12877-020-01678-6)
Supplement: Supplementary file 6 — Additional file 6 Supplementary Table 1. Methodological Quality of included RCT (The Cochrane Collaboration’s tool). It includes the risk of bias of RCTs included in the manuscript. [file 12877_2020_1678_MOESM6_ESM.docx]

| **Supplementary Table 1**. Methodological Quality of the included RCT (The Cochrane Collaboration´s tool). | | | | | | | |
| --- | --- | --- | --- | --- | --- | --- | --- |
| **Study** | **Domain** | | | | | | |
|  | **Random Sequence Generation** | **Allocation Concealment** | **Selective Reporting** | **Blinding of Participants and Personnel** | **Blinding of Outcome Assessment** | **Incomplete Outcome Data** | **Other Bias** |
| Doi et al [56], 2013. | Low risk | Unclear | Unclear | Unclear | Low risk | Low risk | Low risk |
| Donnezan et al [57], 2018. | Unclear | Unclear | Unclear | Unclear | Unclear | Low risk | Low risk |
| Schwenk et al [58], 2016. | Low risk | Unclear | Low risk | High risk | Unclear | Low risk | Low risk |
| Fogarty et al [59], 2016. | Unclear | Unclear | Unclear | Unclear | Unclear | Low risk | Low risk |
| Bae et al [60], 2018. | Low risk | Low risk | Low risk | Low risk | Low risk | Low risk | Low risk |
| Delbroek et al [61], 2017. | Unclear | Unclear | Unclear | Unclear | Unclear | Low risk | Low risk |
| Liao et al [62], 2019. | Unclear | High | Low risk | Low risk | Unclear | Low risk | Low risk |
| **Summary** | **Low: 3**  **High: 0**  **Unclear: 4** | **Low: 1**  **High: 1**  **Unclear: 5** | **Low: 3**  **High: 0**  **Unclear: 4** | **Low: 2**  **High: 1**  **Unclear: 4** | **Low: 2**  **High: 0**  **Unclear: 5** | **Low: 7**  **High: 0**  **Unclear: 0** | **Low: 7**  **High: 0**  **Unclear: 0** |
| In summary, for incomplete outcome data and other bias, all studies had low risk of bias. For random sequence generation, 4 studies did not provide enough detail to determine whether a random sequence generation was used, and 3 studies were identified to have a low risk of bias. For allocation concealment, 1 study had a high risk of bias, 1 study had a low risk of bias, however, 5 studies did not provide enough detail to determine whether an allocated concealmed was employed. For selective reporting and blinding of outcome assessment, 3 studies had a low risk of bias and 4 studies showed an unclear risk of bias. Finally, for blinding of personnel and participants, 2 studies had a low risk of bias; 1 study had a high risk of bias since it was an open-label randomized controlled trial, and 4 studies did not specify if participants and personnel were blinded. | | | | | | | |
