# Supplementary material for: Functional parameters indicative of mild cognitive impairment: a systematic review using instrumented kinematic assessment
Source: BMC Geriatr. 2020 Aug 10;20:282. doi: 10.1186/s12877-020-01678-6 (PMC7418187; doi:10.1186/s12877-020-01678-6)
Supplement: Supplementary file 7 — Additional file 7 Supplementary Table 2. Methodological Quality of included prospective longitudinal studies (The Newcastle Ottawa Scale (NOS)). It shows the risk of bias of included prospective non-randomized longitudinal studies. [file 12877_2020_1678_MOESM7_ESM.docx]

| **Supplementary Table 2.** Methodological Quality of the included prospective longitudinal studies (The Newcastle Ottawa Scale (NOS)). | | | | | |
| --- | --- | --- | --- | --- | --- |
| **Study** | **Domain** | | | | |
|  | **Selection** | **Comparability** | **Exposure and Outcome** | **Total Score** | **Quality** |
| Gillain et al [63], 2015. | 3 | 2 | 2 | 7/9 | Low risk of bias |
| Hayes et al [64], 2008. | 3 | 1 | 2 | 6/9 | Moderate risk of bias |
| Ansai et al [65], 2018. | 3 | 0 | 2 | 5/9 | Moderate risk of bias |
| Dodge et al [67], 2012. | 3 | 1 | 2 | 6/9 | Moderate risk of bias |
| Pieruccini- Faria et al [68], 2018. | 3 | 1 | 2 | 6/9 | Moderate risk of bias |
| Montero-Odasso et al [69], 2009. | 3 | 2 | 2 | 7/9 | Low risk of bias |
| Byun et al [70], 2018. | 3 | 2 | 2 | 7/9 | Low risk of bias |
| Akl et al [71], 2015. | 3 | 2 | 2 | 7/9 | Low risk of bias |
| Akl et al [72], 2015. | 3 | 2 | 2 | 7/9 | Low risk of bias |
| Buchman et al [73], 2019. | 3 | 2 | 2 | 7/9 | Low risk of bias |
| **The risk of bias based on the NOS was classified as: low risk of bias (7-9 points), moderate risk of bias (4-6 points) and high risk of bias (0-3 points).** | | | | | |
