# Supplementary material for: Functional parameters indicative of mild cognitive impairment: a systematic review using instrumented kinematic assessment
Source: BMC Geriatr. 2020 Aug 10;20:282. doi: 10.1186/s12877-020-01678-6 (PMC7418187; doi:10.1186/s12877-020-01678-6)
Supplement: Supplementary file 8 — Additional file 8 Supplementary Table 3. Summary of findings and Quality of evidence assessment (GRADE). It contains the quality of the evidence of each outcome informed at least by two studies with the same design based on the GRADE criteria. [file 12877_2020_1678_MOESM8_ESM.docx]

| **Supplementary Table 3**. Summary of findings and Quality of evidence assessment (GRADE). | | | | | | | | | | |
| --- | --- | --- | --- | --- | --- | --- | --- | --- | --- | --- |
| **Summary of findings** | | | **Quality of evidence assessment (GRADE)** | | | | | | | |
| **Outcome** | **N◦ studies** | **N◦ participants** | **Design** | **Risk of Bias** | **Inconsistency** | **Indirectness** | **Imprecision** | **Other** | **Level of evidence** | **Importance** |
| Walking Speed | 6 | 298 | RCT | Serious^1^ | Not serious^2^ | Some uncertainty^3^ | Serious^4^ | Not reporting bias detected | Low | Critical |
| Walking Speed | 9 | 1662 | Cohort study | Not serious^1^ | Not serious^2^ | Some uncertainty^3^ | Serious^4^ | Not reporting bias detected | Low | Critical |
| Stride time | 3 | 112 | RCT | Serious^1^ | Not serious^2^ | Some uncertainty^3^ | Serious^4^ | Not reporting bias detected | Low | Critical |
| Stride lenght | 4 | 146 | RCT | Serious^1^ | Not serious^2^ | Some uncertainty^3^ | Serious^4^ | Not reporting bias detected | Low | Critical |
| Gait variability | 3 | 112 | RCT | Serious^1^ | Not serious^2^ | Some uncertainty^3^ | Serious^4^ | Not reporting bias detected | Low | Critical |
| TUG | 2 | 89 | RCT | Serious^1^ | Not serious^2^ | Some uncertainty^3^ | Serious^4^ | Not reporting bias detected | Low | Critical |
| Balance | 2 | 62 | RCT | Serious^1^ | Not serious^2^ | Some uncertainty^3^ | Serious^4^ | Not reporting bias detected | Low | Critical |
| Coefficient of variation | 3 | 204 | Cohort study | Not serious^1^ | Not serious^2^ | Some uncertainty^3^ | Serious^4^ | Not reporting bias detected | Low | Critical |
| Gait variability | 3 | 1430 | Cohort study | Not serious^1^ | Not serious^2^ | Some uncertainty^3^ | Serious^4^ | Not reporting bias detected | Low | Critical |
| Activity in home | 3 | 204 | Cohort study | Not serious^1^ | Not serious^2^ | Some uncertainty^3^ | Serious^4^ | Not reporting bias detected | Low | Critical |
| 1. >50% (Not Serious) of the information is from studies with low risk of bias which rarely can affect the interpretation of results. >50% (Serious) or 75% (Very Serious) of the information is from studies with high/moderate risk of bias which sufficiently can affect the interpretation of results.  2. There is not an important unexplained inconsistency in the results. There are not differences in the direction of effect, in the size of the differences in effect, and in the significance of the differences.  3. Differences in intervention or differences in outcome; differences in population.  4. Sample size is small but confidence intervals are not wide; 95% CI is not reported; or sample size is large but the 95% CI around an effect does not exclude 1.0 or 0.0 and there is not a significant effect. | | | | | | | | | | |
